# Protein composition of interband regions in polytene and cell line chromosomes of *Drosophila melanogaster*

**DOI:** 10.1186/1471-2164-12-566

**Published:** 2011-11-18

**Authors:** Sergey A Demakov, Tatyana Yu Vatolina, Vladimir N Babenko, Valery F Semeshin, Elena S Belyaeva, Igor F Zhimulev

**Affiliations:** 1 Institute of Molecular and Cellular Biology, Siberian Branch of Russian Academy of Sciences, Novosibirsk, 630090, Russia; 2 Institute of Cytology and Genetics, Siberian Branch of Russian Academy of Sciences, Novosibirsk, 630090, Russia

## Abstract

**Background:**

Despite many efforts, little is known about distribution and interactions of chromatin proteins which contribute to the specificity of chromomeric organization of interphase chromosomes. To address this issue, we used publicly available datasets from several recent Drosophila genome-wide mapping and annotation projects, in particular, those from modENCODE project, and compared molecular organization of 13 interband regions which were accurately mapped previously.

**Results:**

Here we demonstrate that in interphase chromosomes of *Drosophila *cell lines, the interband regions are enriched for a specific set of proteins generally characteristic of the "open" chromatin (RNA polymerase II, CHRIZ (CHRO), BEAF-32, BRE1, dMI-2, GAF, NURF301, WDS and TRX). These regions also display reduced nucleosome density, histone H1 depletion and pronounced enrichment for ORC2, a pre-replication complex component. Within the 13 interband regions analyzed, most were around 3-4 kb long, particularly those where many of said protein features were present. We estimate there are about 3500 regions with similar properties in chromosomes of *D. melanogaster *cell lines, which fits quite well the number of cytologically observed interbands in salivary gland polytene chromosomes.

**Conclusions:**

Our observations suggest strikingly similar organization of interband chromatin in polytene chromosomes and in chromosomes from cell lines thereby reflecting the existence of a universal principle of interphase chromosome organization.

## Background

Genetic activity of interphase chromosomes is intimately linked to the properties of chromatin organization. At a very basal level, chromatin is organized in nucleosomes, histone octamere/DNA complexes. These, in turn, form higher-order structures, such as chromomeres, loops, domains, etc. Clearly, key to this organization are the chromatin proteins: histones, their post-translational modifications, and non-histone proteins. Modern methods help reliably address the question of interphase chromatin organization at a nucleosomal level, however details of higher-order chromatin organization still remain obscure. This is largely due to our inability to directly visualize the supra-nucleosomal structures in diploid interphase nuclei. Giant polytene chromosomes from dipterans, in particular from *Drosophila*, allow one to mitigate this problem.

"Classic" polytene chromosomes from larval salivary glands of *D. melanogaster *are composed of bundles of one to two thousand tightly synapsed chromosomal strands, which are formed via multiple rounds of endoreplication of just two starting chromatids. As all the homologous chromomeres from all chromatids are aligned to each other, this results in the formation of a thick "cable" with transverse stripes of compacted chromatin (bands) alternating with decompacted interchromomeric regions (interbands). Local differences in size and compaction of banded material form a unique banding pattern that can be used to accurately map any polytene chromosome region. This in turn allows one to link a particular DNA sequence, genes and proteins to the specific chromosomal region, and so to spatially analyze the genetic processes taking place in the interphase nucleus (for review: [1]).

According to different estimates, there are 3500-5000 bands and interbands in *Drosophila melanogaster *polytene chromosomes; these comprise about 95% and 5% of euchromatic DNA, respectively. On average this corresponds to 30 kb of genomic material per band and 2 kb per interband [2-4]. Obviously, the vast majority of genes are situated in bands, as they encompass most of the DNA. As a rule, the degree of chromatin compaction in bands correlates with their transcriptional activity. This is manifested most clearly in case of puffing, i.e. when upon gene activation bands form puffs. Despite the fact that interbands are also represented by decompacted chromatin, their genetic organization and functions are still largely enigmatic. Several hypotheses regarding the functions of interbands were put forward in the literature (for review: [4]), but can be essentially reduced to just two alternatives. Namely, the interbands correspond to active genes. Or, interbands harbor regulatory regions for genes that are found in the neighboring bands. Neither of these scenarios had been adequately addressed experimentally.

In light microscope, many decompacted regions appear as interbands, however upon closer examination at an EM-level they in fact comprise series of faint bands. Thus, of the regions typically considered interband-like, only some are true interbands. Presently it is well-known that numerous "open chromatin" proteins are typically found in such decompacted regions. For instance, these are different forms of RNA polymerase II [5,6], including the paused RNA polymerase II [7,8] which is necessary for transcription initiation; these are proteins and protein complexes involved in transcriptional elongation: SPT4, SPT5, SPT6, TFIIH, dMEDIATOR, dELL [8-11]. Likewise, these regions frequently contain nucleosome remodeling and histone-modifying proteins: CHD1 [12], JIL-1 [13], BRM [14], COHESIN [15], TRX [16], WDS [17], H2B monoubiquitinating enzyme BRE1 [18], and NURF, which increases accessibility of chromatin templates [19]; they harbor histone variants: H4K16ac [20], H3K9ac, H3K14ac [21], H3K4me3 [22]. Furthermore, insulator proteins BEAF-32 [23] and GAF [24] as well as pre-replication complex protein ORC6 [25] are also found in many decompacted regions of polytene chromosomes. Finally, there are at least two interband-specific and interacting proteins, Z4 and CHRIZ (CHROMATOR), however their functions in interbands are presently unknown [26,27].

Despite this plethora of interesting chromatin proteins linked to interbands, their very cytological mapping is not accurate enough, as it is quite challenging to reliably map the protein localization signal to a fine structure of an interband, at least at the resolution level of light microscopy.

**Figure 1:**
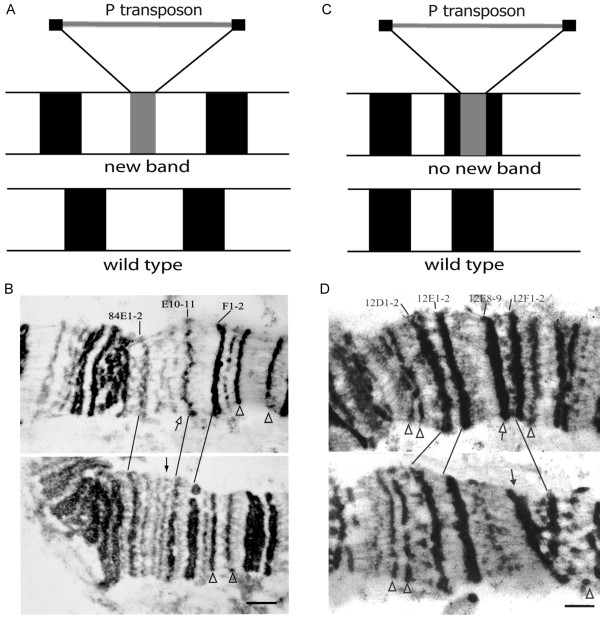
**Morphology of P-element insertions in polytene chromosomes**. Possible scenarios: A - transgenic insertion into the interband results in formation of a novel band; B - electron microscopy image of the region 84E from chromosome arm 3R of wild-type (top) and transgenic for cHBΔ (bottom) larvae. Transgenic material forms a novel band (black arrow), which is absent from the chromosomes in control stock (white arrow); C - transgenic insertion does not result in formation of a novel band; D - electron microscopy image of the region 12E of chromosome X from wild-type (top) and cHBΔ transgenic (bottom) larvae. Chromosome morphology remains unaltered (black arrow) in the transgenic strain as compared to the wild-type chromosome (white arrow). Some marker bands are shown by arrowheads. Bar corresponds to 1 mkm.

Clearly, in order to address the functions of interbands, it is important to be able to accurately map interband regions on a physical map and then to analyze the protein binding profiles and chromatin features in these regions. Unfortunately, using standard mapping techniques, it is close to impossible to precisely map DNA sequences to interbands as their axial lengths are quite small (0.12 mkm on average) [2]. To solve this problem, one must develop new approaches to mark and identify interband regions. P-element insertions could serve as such useful "markers". Using electron microscopy (EM) analysis of polytene chromosomes from stocks with P-element-based insertions, our group has previously shown that such insertions can be visualized on polytene chromosomes as distinct cytological structures [28,29]. In most cases, transcriptionally silent chromatin in such transgenes becomes compacted and forms novel bands, provided that insertions occurred into interbands. When inserted into bands, the compacted material from a transgene typically fuses with the neighboring material and does not form a separate band (Figure 1). As the transgene sequence is known, cloning the DNA sequence adjacent to the transgene insertion is straightforward, and so one can unambiguously identify the sequences that belong to interbands [30-32].

Using this approach, we mapped and cloned the DNA from 13 interband regions. We found that these interbands were mainly composed of non-coding intergenic regions and 5'-UTRs. Also, many of the interbands were rich in DNase I hypersensitive sites (DHSs), which turned out to behave as "hot spots" for integration of P-element based transgenes [33].

With these observations in hands, we decided to further explore the question of functional organization of interbands. First of all, we wanted to establish which proteins were specific to the interbands' open chromatin, and then to ask whether localization of some of these proteins could be correlated on a genome-wide scale. Obviously it was of utmost importance also to understand whether the interbands from polytene chromosomes were "mirrored" by analogous regions in chromosomes from cell lines. Also, in order to address the question of existence of a defined molecular border between bands and interbands, it was interesting and necessary to estimate the length of DNA sequences associated with such proteins. To tackle all these questions, we analyzed the data from *Drosophila *genome-wide protein mapping databases, mostly those from NHGRI modENCODE project [34] and from Filion with co-authors [35]. These projects included comprehensive genome-wide analysis of a wide array of chromatin proteins and histone modifications from *D. melanogaster *cell lines. As a result, 5 [35], 9 and even 30 [36] distinct chromatin types were identified, which were characterized by specific combinations of classes of genes and associated proteins.

Using the abovementioned data obtained on interphase chromosomes of cell lines, in the present work we performed comparative analysis of thirteen interband regions from polytene chromosomes searching for the proteins specifically enriched in interbands. Vast majority of interbands studied was found to associate with a set of proteins that is typically found in open chromatin. These open chromatin proteins tended to localize to low nucleosome density and histone H1-depleted regions and to correlate with binding of ORC2, a pre-replication complex protein. Our data suggest that regions possessing most of these features combined are typically smaller than 3-4 kb in length, and that the number of such regions closely matches the estimated number of cytologically distinct interbands in polytene chromosomes. Furthermore, our data demonstrate that interband chromatin is similarly organized in different cell types, thereby suggesting its participation in general processes that serve to form and maintain the functional architecture of interphase chromosomes.

## Results

### Open chromatin proteins and histone marks are found in the cell line chromosome regions that correspond to polytene chromosome interbands

**Figure 2:**
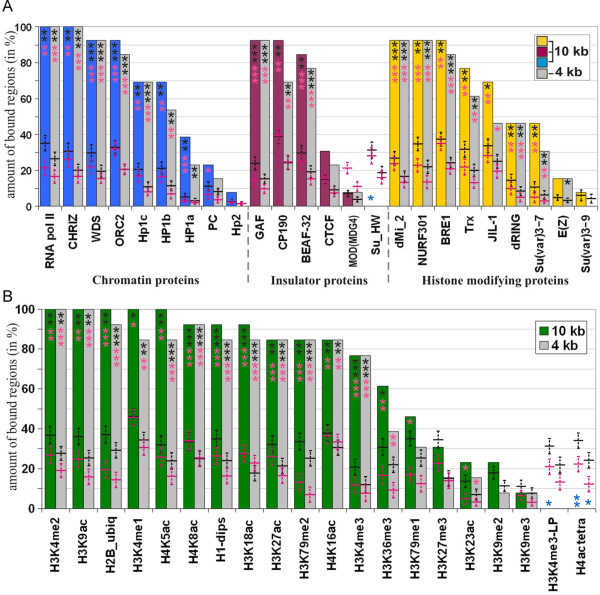
**Integrative view of chromatin proteins distribution over genomic regions of cell lines corresponding to 13 interbands from polytene chromosomes**. A - non-histone proteins; B - histone modifications. Proteins analyzed are shown on the *X *axis, *Y *axis shows percent of interband regions bound by said proteins (out of 13 regions total). Colored bars correspond to percent of regions bound within 10 kb around insertion site; grey bars show percent of regions bound within 4 kb around reference transgenic insertions. Mean and standard deviation for randomly chosen genome regions and band sequences used as a control are represented by the colors black and pink, respectively. * - *P*-value (1,0E-02..1,0E-03), ** - *P*-value (1,0E-03..1,0E-06), *** - *P*-value < 1,0E-06; black and pink shaded asterisks: observed values > expected values, blue shaded asterisks: observed values < expected values.

Distribution profiles for several dozens of proteins and histone marks in several *D. melanogaster *cell types have been established through the efforts of modENCODE project [34]. We used these data and other chromatin features and focused on the regions that correspond to 13 previously mapped interband regions from polytene chromosomes [31,33]. Specifically, we used modENCODE ChIP-chip datasets for S2 cells and in some instances Kc167 cells, which were generated for 18 histone modifications and 25 chromatin proteins belonging to different functional classes. Notably, band/interband transition points remain presently unknown, and interband size estimates also vary quite widely from 0.3 to over 3.8 kb [1,37]. Thus, we compared binding profiles for these proteins over 10 kb regions centered around insertion sites of reference transgenes which were mapped to the interbands studied and used to clone respective DNA sequences (Additional file 1 Figure S1, Additional file 2 Table S1). Figure 2 illustrates that in cell lines most of the 13 regions analyzed (80-100%) associate with open chromatin proteins. Notably, most of these proteins show significantly lower levels of the distribution in control sets of random DNA sequences of equal size from the *D. melanogaster *genome or from three large molecularly mapped bands 10A1-2, 75C1 and 75C2 (Figure 2, Additional file 2 Table S4) [38,39]. Of these open chromatin proteins, RNA polymerase II, CHRIZ, ORC2, GAF, BEAF-32, CP190, TRX, as well as H3K9ac, H4K16ac and H3K4me3 were previously reported to partially or completely immunolocalize to interbands (for review: [4]). The rest of the proteins - WDS, dMI-2, NURF301, BRE1, H3K4me2/3 and H4K16ac were known to contribute to chromatin remodeling and transcriptional regulation. We failed to observe H3K4me3-LP and tetra-H4ac in interband regions, even though these histone marks were reported as present in transcriptionally active chromatin (Supplementary Figures 11-12 from [36]. We attribute this to the quality of H3K4me3-LP antibody: despite H3K4me3 (affinity-purified) and H3K4me3-LP (crude serum) show overall very similar distribution profiles (Additional file 1 Figure S1), the latter antibody rarely displays enrichment above the significance threshold defined by modENCODE.

Another peculiar feature of the regions studied is that they very frequently (> 90%) encompass H1-dips (Figure 2B, Additional file 1 Figure S1) - the regions depleted for histone H1 [40]. This linker histone is known to be the key protein in compacting the 10 nm chromatin fiber into 30 nm super-beaded form [41]. Therefore, presence of H1-dips can be considered as a marker of open chromatin. It is interesting to note that the trends observed for proteins and histone marks associated with open chromatin over 10 kb were essentially the same even over 4 kb centered at insertion points of reference transgenes (Figure 2). This might point to the possible functional interactions of said proteins in these regions of the genome. We next observed that 50-70% of the regions analyzed were also associated with HP1c, HP1b, JIL-1, dRING, H3K36me3 and H3K79me1. Finally, in the regions that correspond to interbands, in cell lines there was no or very little binding for typical "closed chromatin" (transcriptionally inert chromatin) proteins such as HP1a, PC, HP2b, MOD(MDG4), SU(HW), E(Z), SU(VAR)3-7, SU(VAR)3-9, H3K9me2, H3K9me3, H3K27me3, H3K23ac (Figure 2).

**Figure 3:**
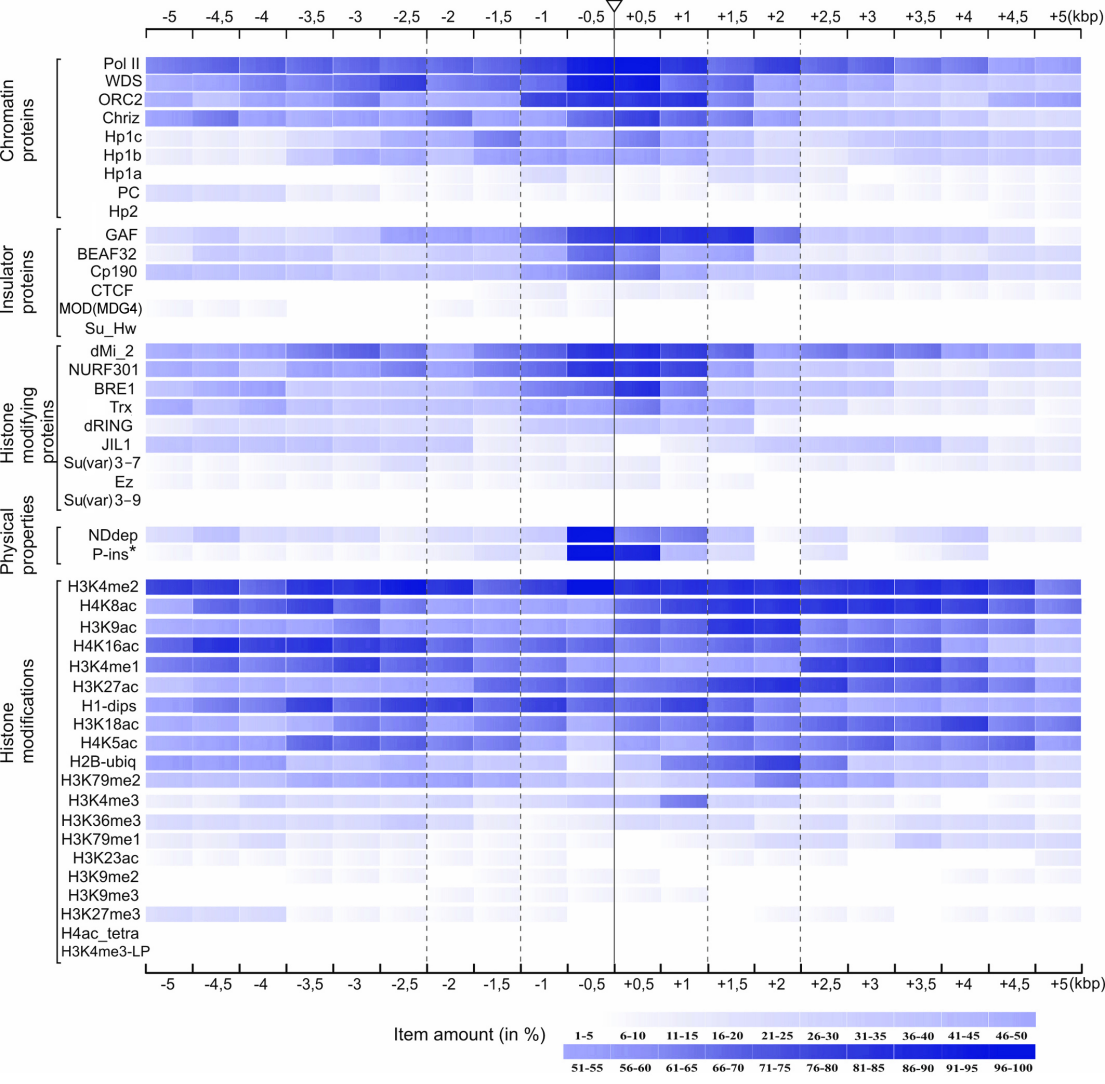
**Heat-map for protein and chromatin features found in 0.5 kb segments over 10 kb regions centered at the insertion sites of reference P-transposons**. Percent of fragments that bind a specific protein or display nucleosome density depletion or harbor P-element insertions is color-coded on the bottom. *Asterisk *-relative content of P-insertions in the segments is shown (see Additional file 2 Table S5). Insertion sites of reference P-transposons are indicated by a solid vertical line. Vertical dashed lines indicate the most likely borders of interbands.

We then analyzed in more detail the profiles for each of the chromatin proteins and histone marks, for P-element insertions and for nucleosome-depleted regions within ± 5 kb from insertion sites of reference transgenes in 13 interband regions. DNA sequences encompassing 1.5-4 kb around these sites were considerably enriched in many open chromatin proteins, such as RNA polymerase II, CHRIZ, ORC2, GAF, BEAF-32, CP190, TRX, WDS, dMI-2, NURF301 and BRE1. Furthermore, these same regions tended to display lower nucleosome density and served as hot spots for P-element integrations (Figure 3, Additional file 1 Figure S1). Of the histone marks that are characteristic of active chromatin, the following five were most frequently (50-100%) and widely (8-10 kb) found: H3K4me2, H4K8ac, H3K9ac, H3K4me1 and H4K16ac. In contrast to non-histone proteins found in active chromatin, the distribution of "active" histone marks is somewhat wider, with slight increase towards the edges of the sequences analyzed (Figure 3). As it was mentioned above, in the interband regions studied, the enrichment for "inactive" marks is close to negligible; hence we failed to identify any peculiar features in their localization.

**Figure 4:**
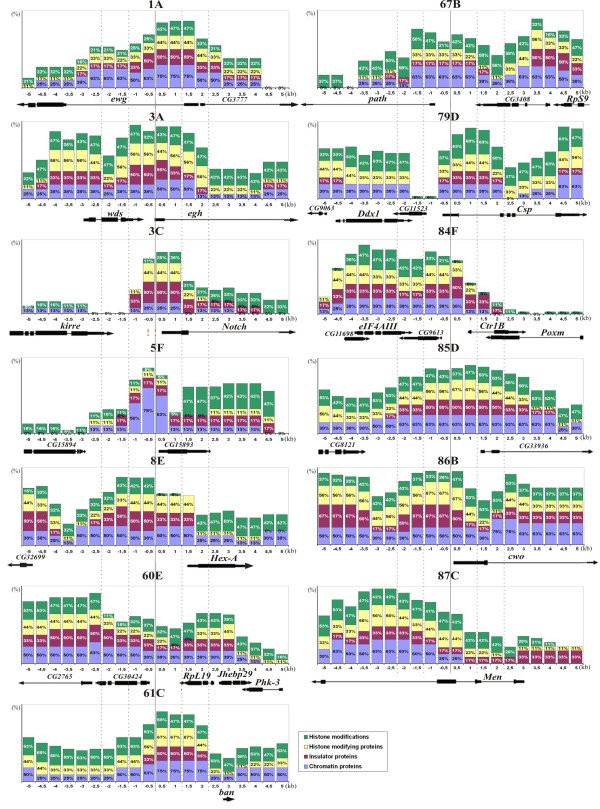
**Distribution of chromatin proteins over the regions corresponding to individual interbands in *D. melanogaster *polytene chromosomes**. *X *axis shows 10 kb of a physical map for the specific region centered at the insertion site of a reference P-transposon (shown as a solid vertical line). Coordinates of the reference transgenic insertions are shown in Additional file 2 Table S1. Position and orientation of underlying genes (as in FlyBase Genes r. 5.12) is indicated below as horizontal blocks and arrows. *Y *axis shows combined percentages of 0.5 kb long DNA segments found associated with a particular class of proteins (n = 20). Color-coding for such classes is indicated below. Vertical dashed lines delimit the regions most probably corresponding to interbands.

Figure 4 demonstrates enrichment profiles for different functional classes of proteins over the regions of interphase chromosomes from cell lines that correspond to polytene chromosome interbands. Histone marks appear either widely enriched or uniformly distributed along the whole region, or slightly increasing towards the ends of the sequences. For most regions, non-histone proteins which mainly comprise markers of active chromatin are enriched over 1.5-4 kb around insertions sites of reference transgenes. However, in two instances, namely in interbands 60E8/E10and 87C8/9, - these enrichment regions are rather found next to the reference insertion sites. We interpret these data as the transgenic insertions hitting the very edge of an interband; alternatively this could be a consequence of distinct transcriptional activities in these regions in salivary glands and in cell lines.

Overall, the data presented here argue in favor of apparent protein-wise similarity in chromatin organization of 13 "true" interband regions studied in polytene chromosomes and of the corresponding regions of genome in cell lines.

### Genome-wide analysis of proteins found in interband regions

To uncover the genome-wide localization characteristics for proteins that map to selected interband regions, we used GEO (Gene Expression Omnibus) datasets available as gff-files at http://www.ncbi.nlm.nih.gov/gds. These files describe genomic regions significantly bound by most of the proteins assayed by modENCODE. We selected fragments with positive scores for non-histone proteins and H1-dips (Additional file 2 Table S3) for all *Drosophila *chromosomes and estimated their genome-wide distributions and lengths of the fragments. Large fraction (70-95%) of these fragments, bound by either "active" or "silent" chromatin proteins, was 1 to 3 kb long (Table 1). The number of fragments bound by "active" chromatin proteins, - RNA polII, CHRIZ, WDS, ORC2, H1-dips, GAF, CP190, BEAF-32, dMI-2, NURF301, BRE1, TRX, -and ranging 1-3 kb, is 3000-5300 (Table 1), which roughly corresponds to the observed number of interbands in polytene chromosomes [4]. On the contrary, there are far fewer fragments (760-2800) that are of similar size (1-3 kb) and are associated with "silent" chromatin proteins PC, E(Z), dRING, or with typical insulator components: CTCF MOD(MDG4), SU(HW) (Table 1).

**Table 1:** Genome-wide analysis of the number and lengths of DNA fragments bound by the proteins represented within interband regions

	Fragment lengths (in kb)			
	
Protein	1-3	4-6	7-9	10-11
	
	Number of fragments (%)			
**RNA pol II**	4760(72.6)	1288(19.6)	396(6.0)	111(1.7)
**CHRIZ**	3045(67.7)	1103(24.5)	264(5.9)	89(2.0)
**WDS**	4639(89.8)	484(9.4)	41(0.8)	4(0.1)
**ORC2**	4178(84.4)	651(13.1)	108(2.2)	16(0.3)
**H1_dip**	3543(76.3)	796(17.1)	237(5.1)	68(1.5)
**PC**	1491(72.6)	373(18.2)	138(6.7)	52(2.5)
**GAF**	2909(80.2)	591(16.3)	110(3.0)	16(0.4)
**CP190**	4982(86.1)	714(12.3)	81(1.4)	11(0.2)
**BEAF-32**	4060(88.0)	499(10.8)	53(1.1)	4(0.1)
**CTCF**	1446(75.3)	405(21.1)	62(3.2)	7(0.4)
**MOD(MDG4)**	843(98.9)	9 (1.1)	0(0.0)	0(0.0)
**Su(Hw)**	2829(69.6)	1020(25.1)	178(4.4)	36(0.9)
**dMi-2**	3802(83.2)	625(13.7)	118(2.6)	23(0.5)
**NURF301**	5336(87.0)	680(11.1)	101(1.6)	13(0.2)
**BRE1**	5245(87.7)	675(11.3)	54(0.9)	7(0.1)
**Trx-C**	4885(93.6)	319(6.1)	16(0.3)	0(0.0)
**dRing**	2019(88.4)	198(8.7)	55(2.4)	13(0.6)
**E(Z)**	760(83.6)	113(12.4)	27(3.0)	9(1.0)

**Figure 5:**
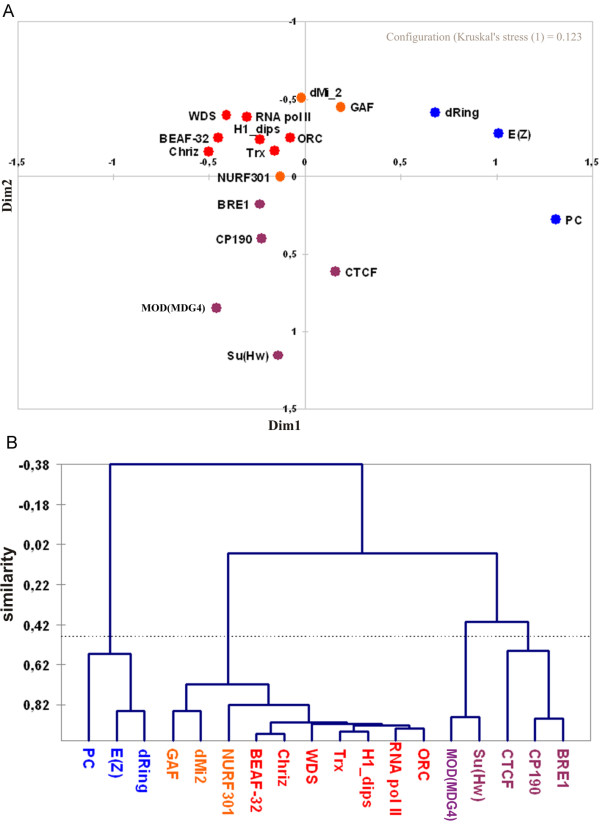
**Graphic representation of co-localization extent for "interband" chromatin proteins over the entire fly genome**. A - Multidimensional scaling (MDS) plot for 18 binding factors; Horizontal and vertical axes show the degree of co-localization in conditional units. B - Agglomerative hierarchical clustering (AHC) of factors analyzed (XLStat Inc, http://www.xlstat.com).

In order to estimate how frequently these proteins co-localize in *D. melanogaster *genome, we performed their pair-wise comparison. The number of overlapping pairs was considered as a similarity measure for every pair of factors being compared. Only the fragments that showed positive scores and which were smaller than 10 kb were considered. We calculated the number of unique paired overlaps between the fragments (Additional file 2 Table S6) and so estimated the pair-wise correlation coefficients between the proteins (Additional file 2 Table S7). The highest values of correlation coefficients were observed for the "active" chromatin proteins and for proteins enriched in 13 interbands, i.e. for BEAF-32, CHRIZ, RNA POL II, ORC2, H1-dips, TRX, WDS, NURF301 and BRE1. The same was observed for "silent" chromatin group of proteins - MOD(MDG4), SU(HW), E(Z), dRING. To verify whether this co-localization is significant, we first fragmented the euchromatic part of the genome (120 Mb) into non-overlapping 3 kb-long blocks (the median size of fragments that are bound by these proteins (Table 1)). Then we analyzed each of these ~40000 blocks for the presence of all pair-wise combinations of these proteins. As it is shown in Additional file 2 Table S8, the probability of independent pair-wise localization of all "active" proteins in interbands studied is fairly low (*P*-value < 10^-300^). Figure 5A shows a multidimensional scaling plot (see Methods) of the correlations mentioned above. The "active" chromatin proteins characteristic of interbands cluster together and away from the cluster of "silent" chromatin proteins that do not map to interbands.

Using the agglomerative hierarchical clustering (AHC) approach, we estimated the co-localization frequencies for all the proteins. These formed 3 separate groups (Figure 5B). First group comprised the "active" chromatin factors, such as BEAF-32, CHRIZ, H1-dips, RNA polII, ORC2, TRX and WDS, many of which were reported to immunolocalize to decompacted regions of polytene chromosomes. It is interesting to note that the numbers of pair-wise overlaps for the proteins from this group are fairly tight, ranging from 3300 to 3800 (3600 on average), which fits very well the number of interbands in polytene chromosomes [4]. Nucleosome remodeling proteins such as NURF301, dMI-2 and GAF also tend to co-localize with this group. The two remaining groups of proteins are represented mainly by Pc-G proteins - PC, E(Z), dRING and by insulator proteins, MOD(MDG4), SU(HW), CTCF, CP190, and surprisingly by BRE1. These proteins display low levels of co-localization frequency with the proteins from the first group, and so appear not to be present in interbands.

## Discussion

**Figure 6:**
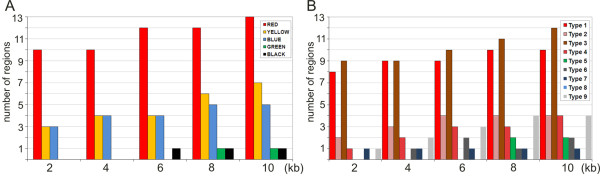
**Distribution of various chromatin states in 13 regions of *D. melanogaster *genome that correspond to interbands in polytene chromosomes**. A - 5 "colored" chromatin states according to [35]; B - 9 chromatin states according to [36]. *X *axis shows sizes of DNA segments centered at the insertion sites of reference P-transposons. *Y *axis shows number of regions associated with a particular type of chromatin.

Using genome-wide distribution data for a wide range of non-histone proteins and histone marks available for *D. melanogaster *cell lines [35,36,40], we analyzed the protein composition and chromatin features in genomic regions of cell line chromosomes corresponding to 13 interband regions of polytene chromosomes. Our results establish these regions as depleted for the linker histone H1 (showing H1 dips), and associated with a specific set of proteins characteristic of "active" chromatin (Figures 2 and 3). This is also consistent with the distribution of different states of chromatin in these genomic regions (Figure 6, Additional file 1 Figure S1). Namely of the five principle states of chromatin that were previously identified in *Drosophila *cell lines and color-coded by Filion with co-authors [35], it is predominantly RED chromatin that we observe most frequently within 10 kb fragments encompassing interbands. This chromatin is reported as enriched in ORC binding sites as well as in regulatory sequences and mainly comprises genes which are linked to specific processes such as "receptor binding", "defense response", "transcription factor activity" and "signal transduction" [35]. The interband regions studied also contain YELLOW and BLUE chromatin (Figure 6A). Transcriptionally active YELLOW chromatin is specifically marked with H3K36me3, a mark of transcriptional elongation typically present on genes with a broad expression pattern over many developmental stages and tissues, so-called "house-keeping" genes. BLUE chromatin is mostly found in genome regions associated with Pc-G proteins and harboring developmental genes as well as many of the highly conserved non-coding elements (HCNEs) that contribute to gene regulation [35]. It is important to emphasize that the fraction of RED chromatin relatively to the rest of the chromatin types increases closer to the insertion sites marking interband regions (Additional File 3 Figure S2). At a 10 kb level, RED chromatin is 1.9 and 2.6 times enriched compared to the YELLOW and BLUE states, respectively, whereas when the regions ± 1 kb around insertion sites are considered, RED chromatin is 3.3 time more frequent. GREEN and BLACK chromatin states characteristic of genetically silent material (pericentric heterochromatin and transcriptionally inactive regions scattered over the genome, respectively) are very rarely found in interbands and if present tend to be located on the flanks (Figure 6A, Additional File 3 Figure S2A).

According to the 9-state model of chromatin organization in cell lines [36], the regions corresponding to interbands are mostly composed of state 1 and state 3 chromatin (Figure 6B, Additional File 3 Figure S2B). State 1 chromatin is rich in promoters, TSSes and 5'-UTRs. State 3 chromatin is mainly characterized by the presence of large first introns in long genes, enrichment for specific chromatin remodeling factors (for instance SPT16 and dMI-2), presence of enhancers and early origins of replication. As compared to states 1 and 2, state 3 domains show stronger enrichment for transcription-associated histone variant H3.3 [36]. Despite some differences in approaches as well as in the proteins analyzed in [35,36], the regions that correspond to 13 interbands display consistent set of features. They are mostly represented by regulatory and promoter regions for the genes which appear to reside in the adjacent compacted material of bands (chromomeres).

Most of the "active" chromatin proteins that mapped in cell lines to DNA regions corresponding to interbands, are known to immunolocalize to interbands (for review: [4]). Therefore, it is plausible to suggest that the "open" chromatin feature and the localization of a specific set of proteins are inter-related, and in fact represent a universal principle of interphase chromosome organization. This conclusion is consistent with the highly detailed observations by W. Beermann, who compared banding patterns in four larval tissues of *Chironomus*, and who observed them to match perfectly except for minor differences at certain regions and differences due to puffing [2]. Similar work on *Drosophila *also described very minor changes in banding pattern [42]. Significant similarity in banding patterns was subsequently observed upon comparison of many different tissues from many insects (for review: [1,43]). That "active" chromatin is invariably present in interbands, is also supported by the similar pattern of DHSs in salivary gland polytene chromosomes and in embryonic cells. For instance, mapping of major DHSs on physical and cytological maps of the *fa^swb ^*interband demonstrated their identical localization, length and number in the chromatin of embryonic cells, cell lines [44] and in larval cells [33]. This might help to explain high frequency of P-element integrations into interbands, as insertions tend to hit the regions of DHSs [32,33]. It must be emphasized that P elements transpose and integrate in diploid germline cells, and there are no reasons to believe that insertions sites are linked in any way to the gene expression nearby [45]. Within reference interbands, we observed P-elements to predominantly cluster around open chromatin regions (Figure 3), therefore this might suggest that these same DNA sequences are also organized in open chromatin in germline cells, where P-elements actually transpose and integrate.

## Conclusions

Based on the genome-wide protein mapping data generated by modENCODE on *D. melanogaster *cell lines, and using previously mapped interband regions as a reference, we for the first time demonstrated that decompacted chromatin regions that appear as interbands in polytene chromosomes are organized the same way in other cell types and correspond to interchromomeres of interphase chromosomes in cell lines. The peculiarities of protein distribution identified for interband regions can serve as convenient markers to precisely map interbands to the molecular map, thereby allowing one to compile comparative molecular and cytogenetic maps of interphase chromosomes in different *Drosophila *cell types. Indeed, further experimental validation of band and interband regions on a larger scale should be helpful to firmly establish this conclusion. Using our approach, precise mapping of the band/interband positions across entire *Drosophila *genome is a subject of separate work which is currently underway.

## Methods

### Cytological Analysis of Polytene Chromosomes

Salivary gland polytene chromosome squashes were prepared for electron microscopy analysis and examined as described earlier [46]. The sections with a thickness of 120-150 nm were cut using an LKB-IV ultratome (Sweden) and examined with a JEM-100C (Japan) electron microscope at 80 kV. Transgenic fly stocks contain insertions of cHBΔ transposon, which is an 18 kb-long *P*-transposon encompassing *D. melanogaster *gene *rosy *and β-*gal *from *E. coli *[47].

### Genomic analysis

ChIP-chip data files for chromatin proteins and histone modifications from *Drosophila *cell lines (Additional file 2 Table S2) were downloaded from modENCODE consortium website [48]. The coordinates of chromatin domains determined elsewhere [35] were extracted from NCBI Gene Expression Omnibus [49], accession number GSE22069. Centers of 12 interbands (dm3 assembly) coincided with the integration sites of P transposons used to map respective interbands; for the interband 3C6/C7, proximal border of deletion *fa^swb ^*[50] was selected as a central point. The coordinates of P-transposon insertion sites (Additional file 2 Table S1) were downloaded from FlyBase [51] (release FB2010_01).

To check whether 18 proteins might cluster throughout the whole genome, we performed pair-wise comparison of these regions and counted the number of overlapping pairs as a similarity measure for every pair of binding regions. Only the fragments with positive scores shorter than 10 kb were considered (Additional file 2 Table S3). The formalized procedure was as follows: Let *L_i _*= (*l*_1*i*_, ... *l*_*mi*_), *L_j _*= (*l*_1*j*_, ... *l*_*ni*_) be the vectors representing two binding proteins *i *and *j*; *i, j *∈ [1, ... 18], *m < n *are dimensions (sizes) of the vectors. We remove the redundant regions from *L_1_, L_2 _*which bind the same region from the counterpart vector, thereby obtaining the reduced sizes *m', n' *of the corresponding vectors. We define regions *l_fi _*and *l_hj _*overlap if they possess nonzero common location on DNA. Then we define the similarity rate as $r_{ij}=\frac{k}{\min\left(n^{\prime },m^{\prime }\right)}$, where *k *is the number of overlapping regions, and consequently compile similarity matrix *R = {r_ij_}*. Then we apply multidimensional scaling (MDS) with XLStat add-on software http://www.xlstat.com for the matrix *R *obtained as described at the previous step. We used non-metric MDS model, where only the order of the similarities counts (ordinal (2)).

Agglomerative hierarchical clustering (AHC) with the same metric as in MDS was used to assess non-random clusters in the pair-wise comparisons (XLSTAT Inc).

To evaluate the significance of protein binding sites co-localization, we used chi-square test for 2 × 2 contingency table as follows. We considered the number of non-overlapping fragments with average length about 3 kb in 120 Mb of the eukaryotic part of *D. melanogaster *genome, so we obtained *n = *40000 fragments in total. Next, for each pair of proteins we calculated the contingency table, where *l *and *m - *numbers of peaks with positive scores for the proteins in a pair. The expected (theoretical) number of overlapping sites given random overlap model calculated for two proteins is en = l*m/n. This model is robust to the variance of the total segments in the interval [40000-80000] with significance increasing with increasing total segments model. Thus, we used the value of 40000 as a conservative estimate.

### Statistical analysis

To assess whether protein binding sites preferentially localize to the experimentally confirmed 13 interbands at a statistically significant level, we performed 13000 random samplings of equivalent DNA chunks (4 and 10 kb segments) across *D. melanogaster *genome and calculated the number of corresponding protein binding sites that overlapped with the random regions. The sampling procedure accounts for the observed biases in chromosome localization of the 13 validated interbands (one on chr2R; 3 on chr3L, 4 on chr3R; and 5 on chrX) and uses corresponding weights when selecting random fragments from a chromosome arm. Only binding regions with positive scores were considered. No limitation on the size of a binding site has been imposed. Only single hits per random region were considered. We then calculated the probability of getting a random DNA region of a given size equivalent to the source set (4/10 kb). Thus, we were able to estimate how many of the 13 randomly chosen fragments shall overlap with the given protein binding sites by chance. Also we calculated *P*-value of the observed overlap of the experimentally verified 13 interband regions with the given sets of protein binding sites using Binomial test as follows:

$$P\left(p,m,13\right)=\overset{m}{\underset{i=0}{\sum }}C_{13}^{i}p^{i}{\left(1-p\right)}^{13-i},$$

where *p *- expected by random chance frequency of a given set of protein binding sites to overlap with the DNA region of a given size (4/10 kb), *m *- number of the observed DNA regions that overlap with the given protein binding site set, $C_{13}^{i}$ is a binomial coefficient.

The tail of the binomial distribution to be summed up was chosen based on the observed number of "successes" *m*, which could be either less or more than 13*p. In the case *m >*13*p, we set *P' = 1-P*, otherwise the original *P *was used.

We similarly estimated the expected numbers of regions that associate with the set of proteins studied in 13000 randomly generated DNA chunks of equivalent size (4 and 10 kb) from three molecularly mapped bands: 10A1-2 (ChrX: 108000000-10980000) [38], 75C1 (Chr3L: 18170000-18370000) and 75C2 (Chr3L: 18450000-18610000) [39]. The coordinates of band DNA sequences are shown according to FlyBase [51] (release 5.18).

## Authors' contributions

SAD, ESB and IFZ conceived the study and participated in its design. SAD and VFS carried out electron microscopy analysis. VNB and TYV contributed to bioinformatics analysis. SAD and IFZ wrote the manuscript. All authors read and approved the final manuscript.

## Supplementary Material

Additional file 1 **Figure S1. Localization of proteins and DNA elements around 13 interband regions of cell lines chromosomes**. *Top*: molecular and genetic maps (20 kb) of these regions are centered at positions (solid vertical lines) of reference transposons (triangles) that were used for cytological identification and cloning the DNA around reference transposons in interbands. Exact molecular coordinates of transposon insertions are given in Additional File 2 Table S1. Horizontal arrows denote positions and orientation of known genes (FlyBase Genes r. 5.12). Vertical red arrows correspond to P-transposon integration sites referenced in FlyBase (when insertion sites were too close, their number is indicated above the arrow). For the region 3C6/C7, P-element integration regions lacking precise molecular localization are denoted by horizontal lines; *fa^swb ^*deletion is shown as square brackets. *Bottom*: data on the densities of nucleosomes, distributions of 9 chromatin states and binding sites for chromatin proteins in S2 cell line as presented on the modENCODE website [48], as well as distributions of histone H1 depleted regions (H1-dips) according to [40], the five-colored chromatin types [35] and binding sites for ORC proteins according to [52] in Kc167 cell line. Regions most likely corresponding to interbands are delimited by vertical dashed lines. Click here for file

Additional file 2 **Tables S1-S8**. Supplemental **Tables 1-8**. **Table S1 **Molecular coordinates of integration sites of P-transgenes used to map interbands. **Table S2 **Accession numbers of chromatin proteins. **Table S3 **List of proteins analyzed and number of regions with positive scores. **Table S4 **Frequencies of protein localization in 13 interband regions and in random DNA samplings of *D. melanogaster *genome and band sequences. **Table S5 **Distribution of P transposon insertions within interband regions. **Table S6 **Number of pair-wise overlaps between DNA fragments bound by the chromatin proteins analyzed. **Table S7 **Pair-wise correlation scores for proteins analyzed. **Table S8 **P-value scores for pair-wise correlations between DNA fragments associated with the chromatin proteins analyzed. Click here for file

Additional file 3 **Figure S2. Frequency of chromatin states in 13 regions of *D. melanogaster *genome that correspond to interbands in polytene chromosomes**. A - 5 "colored" chromatin states according to [35]; B - 9 chromatin types according to [36]. Sizes of DNA segments centered at the insertion sites of reference P-transposons *(X *axis); Percentage of DNA fragments associated with a particular type of chromatin calculated for each segment (*Y *axis). Click here for file

## References

[B1] Zhimulev IF Morphology and structure of polytene chromosomes Adv Genet 1996 34 1 497 9348397 10.1016/s0065-2660(08)60533-7

[B2] Beermann W Beermann W Chromomeres and genes Results and problems in cell differentiation 1972 4 Berlin, Heidelberg, New York: Springer 1 33 4198831 10.1007/978-3-540-37164-9_1

[B3] Zhimulev IF Belyeva ES Semeshin VF Informational content of polytene chromosome bands and puffs CRC Crit Rev Biochem 1981 11 303 340 6171381 10.1080/10409238109104420

[B4] Zhimulev IF Belyaeva ES Semeshin VF Koryakov DE Demakov SA Polytene Chromosomes: 70 Years of Genetic Research Int Rev Cytol 2004 241 203 275 15548421 10.1016/S0074-7696(04)41004-3

[B5] Jamrich M Greenleaf AL Bautz EK Localization of RNA polymerase in polytene chromosomes of *Drosophila melanogaster* Proc Natl Acad Sci USA 1977 74 079 83 10.1073/pnas.74.1.79 PMC431078 405671

[B6] Sass H Bautz EKF Interbands of polytene chromosomes: binding sites and start points for RNA polymerase Chromosoma 1982 86 77 93 10.1007/BF00330731 6756817

[B7] Weeks JR Hardin SE Shen J Lee JM Greenleaf AL Locus-specific variation in phosphorylation state of RNA polymerase II in vivo: correlation with gene activity and transcript processing Genes Dev 1993 7 2329 2344 10.1101/gad.7.12a.2329 8253380

[B8] Kaplan CD Morris JR Wu C Winston F Spt5 and spt6 are associated with active transcription and have characteristics of general elongation factors in *D. melanogaster* Genes Dev 2000 14 2623 2634 10.1101/gad.831900 11040216 PMC316994

[B9] Gerber M Ma J Dean K Eissenberg JC Shilatifard A *Drosophila *ELL is associated with actively elongating RNA polymerase II on transcriptionally active sites in vivo EMBO J 2001 20 21 6104 6114 10.1093/emboj/20.21.6104 11689450 PMC125687

[B10] Lis JT Mason P Peng J Price DH Werner J P-TEFb kinase recruitment and function at heat shock loci Genes Dev 2000 14 792 803 10766736 PMC316500

[B11] Park JM Gim BS Kim JM Yoon JH Kim HS *Drosophila *Mediator complex is broadly utilized by diverse gene-specific transcription factors at different types of core promoters Mol Cell Biol 2001 21 7 2312 2323 10.1128/MCB.21.7.2312-2323.2001 11259581 PMC86865

[B12] Stokes DG Tartof KD Perry RP CHD1 is concentrated in interbands and puffed regions of *Drosophila *polytene chromosomes Proc Natl Acad Sci USA 1996 93 7137 7142 10.1073/pnas.93.14.7137 8692958 PMC38949

[B13] Jin Y Wang Y Walker DL Dong H Conley C Johansen J Johansen KM JIL-1: a novel chromosomal tandem kinase implicated in transcriptional regulation in *Drosophila* Mol Cell 1999 4 129 135 10.1016/S1097-2765(00)80195-1 10445035

[B14] Armstrong JA Papoulas O Daubresse G Sperling AS Lis JT The *Drosophila *BRM complex facilitates global transcription by RNA polymerase II EMBO J 2002 21 5245 5254 10.1093/emboj/cdf517 12356740 PMC129039

[B15] Markov AV Zakharov AA Galkin AP Strunnikov AV Smirnov AF Cohesin complexes in polytene chromosomes of *Drosophila melanogaster *are located in interbands Genetika 2003 39 9 1203 1211 (in Russian) 14582389

[B16] Tariq M Nussbaumer U Chen Y Beisel C Paro R Trithorax requires Hsp90 for maintenance of active chromatin at sites of gene expression Proc Natl Acad Sci USA 2009 106 1157 1162 10.1073/pnas.0809669106 19144915 PMC2633526

[B17] Raja SJ Charapitsa I Conrad T Vaquerizas JM Gebhardt P The nonspecific lethal complex is a transcriptional regulator in *Drosophila* Mol Cell 2010 38 6 827 41 10.1016/j.molcel.2010.05.021 20620954

[B18] Tenney K Gerber M Ilvarsonn A Schneider J Gause M *Drosophila *Rtf1 functions in histone methylation, gene expression, and Notch signaling Proc Natl Acad Sci USA 2006 103 32 11970 11974 10.1073/pnas.0603620103 16882721 PMC1567682

[B19] Carré C Ciurciu A Komonyi O Jacquier C Fagegaltier D Pidoux J Tricoire H Tora L Boros IM Antoniewski C The *Drosophila *NURF remodelling and the ATAC histone acetylase complexes functionally interact and are required for global chromosome organization EMBO reports 2007 9 187 192 18084186 10.1038/sj.embor.7401141 PMC2246414

[B20] Lavender JS Birley AJ Palmer MJ Kuroda MI Turner BM Histone H4 acetylated at lysine 16 and other components of the *Drosophila *dosage compensation pathway colocalize on the male X through mitosis Chromosome Res 1994 2 398 404 10.1007/BF01552799 7981944

[B21] Nowak SJ Corces VG Phosphorylation of histone H3 correlates with transcriptionally active loci Genes Dev 2000 14 3003 3013 10.1101/gad.848800 11114889 PMC317109

[B22] Sedkov Y Cho E Petruk S Cherbas L Smith ST Methylation at lysine 4 of histone H3 in ecdysone-dependent development of *Drosophila* Nature 2003 426 6962 78 83 10.1038/nature02080 14603321 PMC2743927

[B23] Zhao K Hart CM Laemmli UK Visualization of chromosomal domains with boundary element-associated factor BEAF-32 Cell 1995 81 879 89 10.1016/0092-8674(95)90008-X 7781065

[B24] Raff JW Kellum R Alberts B The *Drosophila *GAGA transcription factor is associated with specific regions of heterochromatin throughout the cell cycle EMBO J 1994 13 5977 5983 7813435 10.1002/j.1460-2075.1994.tb06943.x PMC395573

[B25] Balasov M Huijbregts RPH Chesnokov I Role of the Orc6 Protein in Origin Recognition Complex-Dependent DNA Binding and Replication in *Drosophila melanogaster* Mol Cell Biol 2007 27 8 3143 3153 10.1128/MCB.02382-06 17283052 PMC1899928

[B26] Eggert H Gortchakov A Saumweber H Identification of the *Drosophila *interband-specific protein Z4 as a DNA-binding zinc-finger protein determining chromosomal structure Cell Sci 2004 117 4253 4264 10.1242/jcs.01292 15292401

[B27] Gortchakov AA Eggert H Gan M Mattow J Zhimulev IF Saumweber H Chriz, a chromodomain protein specific for the interbands of *Drosophila melanogaster *polytene chromosomes Chromosoma 2005 114 54 66 10.1007/s00412-005-0339-3 15821938

[B28] Semeshin VF Belyaeva ES Zhimulev IF Lis JT Richards G Bourouis M Electron microscopical analysis of *Drosophila *polytene chromosomes. IV. Mapping of morphological structures appearing as a result of transformation of DNA sequences into chromosomes Chromosoma 1986 93 461 468 10.1007/BF00386785

[B29] Semeshin VF Demakov SA Perez Alonso M Belyaeva ES Bonner JJ Zhimulev IF Electron microscopical analysis of *Drosophila *polytene chromosomes. V. Characteristics of structures formed by transposed DNA segments of mobile elements Chromosoma 1989 97 396 412 10.1007/BF00292767 2541983

[B30] Demakov SA Semeshin VF Zhimulev IF Cloning and molecular genetic analysis of *Drosophila melanogaster *interband DNA Mol Gen Genet 1993 238 437 443 10.1007/BF00292003 8388080

[B31] Demakov S Gortchakov A Schwartz Y Semeshin V Campuzano S Modolell J Zhimulev I Molecular and genetic organization of *Drosophila melanogaster *polytene chromosomes: evidence for two types of interband regions Genetica 2004 122 311 324 10.1007/s10709-004-2839-0 15609554

[B32] Semeshin VF Demakov SA Shloma VV Vatolina TY Gorchakov AA Zhimulev IF Interbands behave as decompacted autonomous units in *Drosophila melanogaster *polytene chromosomes Genetica 2008 132 267 79 10.1007/s10709-007-9170-5 17657571

[B33] Vatolina TYu Demakov SA Semeshin VF Makunin IV Babenko VN Identification and molecular genetic characterization of the polytene chromosome interbands in *Drosophila melanogaster* Russian Journal of Genetics 2011 47 5 15 26 21786665

[B34] Celniker SE Dillon LA Gerstein MB Gunsalus KC Henikoff S Unlocking the secrets of the genome Nature 2009 459 927 930 10.1038/459927a 19536255 PMC2843545

[B35] Filion GJ van Bemmel JG Braunschweig U Talhout W Kind J Systematic protein location mapping reveals five principal chromatin types in *Drosophila *cells Cell 2010 143 212 224 10.1016/j.cell.2010.09.009 20888037 PMC3119929

[B36] Kharchenko PV Alekseyenko AA Schwartz YB Minoda A Riddle NC Comprehensive analysis of the chromatin landscape in *Drosophila melanogaster* Nature 2011 471 7339 480 4855 10.1038/nature09725 21179089 PMC3109908

[B37] Rykowski MC Parmelee SJ Agard DA Sedat JW Precise determination of molecular limits of polytene chromosome band: regulatory sequences for the Notch gene are in the interband Cell 1988 54 461 472 10.1016/0092-8674(88)90067-0 3135939

[B38] Kozlova TYu Semeshin VF Tretyakova IV Kokoza EB Pirrotta V Grafodatskaya VE Belyaeva ES Zhimulev IF Molecular and cytogenetical characterization of the 10A1-2 band and adjoining region in the *Drosophila melanogaster *polytene X chromosome Genetics 1994 136 1063 1073 8005415 10.1093/genetics/136.3.1063 PMC1205863

[B39] Andreyenkova NG Kokoza EB Semeshin VF Belyaeva ES Demakov SA Pindyurin AV Andreyeva EN Volkova EI Zhimulev IF Localization and characteristics of DNA underreplication zone in the 75C region of intercalary heterochromatin in *Drosophila melanogaster *polytene chromosomes Chromosoma 2009 118 747 761 10.1007/s00412-009-0232-6 19685068

[B40] Braunschweig U Hogan GJ Pagie L van Steensel B Histone H1 binding is inhibited by histone variant H3.3 The EMBO J 2009 28 3635 3645 10.1038/emboj.2009.301 PMC2790488 19834459

[B41] Allan J Mitchell T Harborne N Boehm L Crane-Robinson C Roles of H1 domains in determining higher order chromatin structure and H1 location J Mol Biol 1986 187 591 601 10.1016/0022-2836(86)90337-2 3458926

[B42] Zhimulev IF Belyaeva ES Variation in the banding pattern of the polytene chromosomes of *Drosophila melanogaster *larvae Genetika 1977 13 1398 1408 (in Russian)

[B43] Zhimulev IF Genetic organization of polytene chromosomes Adv Genet 1999 39 1 599 10319426 10.1016/s0065-2660(08)60476-9

[B44] Vasquez J Schedl P Deletion of an insulator element by the mutation facet-strawberry in *Drosophila melanogaster* Genetics 2000 155 1297 1311 10880489 10.1093/genetics/155.3.1297 PMC1461175

[B45] Bellen HJ Levis RW Liao G He Y Carlson JW Tsang G Evans-Holm M Hiesinger PR Schulze KL Rubin GM The BDGP gene disruption project: single transposon insertions associated with 40% of *Drosophila *genes Genetics 2004 167 761 781 10.1534/genetics.104.026427 15238527 PMC1470905

[B46] Semeshin VF Belyaeva ES Shloma VV Zhimulev IF Electron microscopy of polytene chromosomes Methods Mol Biol 2004 247 305 324 14707355 10.1385/1-59259-665-7:305

[B47] Simon JA Sutton CA Lis JT Localization and expression of transformed DNA sequences within heat shock puffs of *Drosophila melanogaster* Chromosoma 1985 93 26 30 10.1007/BF01259442 3933923

[B48] modENCODE consortium http://www.modENCODE.org

[B49] NCBI Gene Expression Omnibus http://www.ncbi.nlm.nih.gov/gds/

[B50] Ramos RGP Grimwade BG Wharton KA Scottgale TN Artavanis-Tsakonas S Physical and functional definition of the *Drosophila *Notch locus by P element transformation Genetics 1989 123 337 348 2555253 10.1093/genetics/123.2.337 PMC1203805

[B51] Tweedie S Ashburner M Falls K Leyland P McQuilton P Marygold S Millburn G Osumi-Sutherland D Schroeder A Seal R FlyBase:enhancing *Drosophila *Gene Ontology annotations Nucleic Acids Res 2009 37 D555 559 10.1093/nar/gkn788 18948289 PMC2686450

[B52] MacAlpine HK Gordân R Powell SK Hartemink AJ MacAlpine DM *Drosophila *ORC localizes to open chromatin and marks sites of cohesin complex loading Genome Res 2010 20 201 211 10.1101/gr.097873.109 19996087 PMC2813476

