# Supplementary material for: Protein composition of interband regions in polytene and cell line chromosomes of Drosophila melanogaster
Source: BMC Genomics. 2011 Nov 18;12:566. doi: 10.1186/1471-2164-12-566 (PMC3240664; doi:10.1186/1471-2164-12-566)
Supplement: Additional file 1 — Figure S1. Localization of proteins and DNA elements around 13 interband regions of cell lines chromosomes. Top: molecular and genetic maps (20 kb) of these regions are centered at positions (solid vertical lines) of reference transposons (triangles) that were used for cytological identification and cloning the DNA around reference transposons in interbands. Exact molecular coordinates of transposon insertions are given in Additional File 2 Table S1. Horizontal arrows denote positions and orientation of known genes (FlyBase Genes r. 5.12). Vertical red arrows correspond to P-transposon integration sites referenced in FlyBase (when insertion sites were too close, their number is indicated above the arrow). For the region 3C6/C7, P-element integration regions lacking precise molecular localization are denoted by horizontal lines; faswb deletion is shown as square brackets. Bottom: data on the densities of nucleosomes, distributions of 9 chromatin states and binding sites for chromatin proteins in S2 cell line as presented on the modENCODE website [48], as well as distributions of histone H1 depleted regions (H1-dips) according to [40], the five-colored chromatin types [35] and binding sites for ORC proteins according to [52] in Kc167 cell line. Regions most likely corresponding to interbands are delimited by vertical dashed lines. [file 1471-2164-12-566-S1.PDF]

Region 1A8/1B1-2

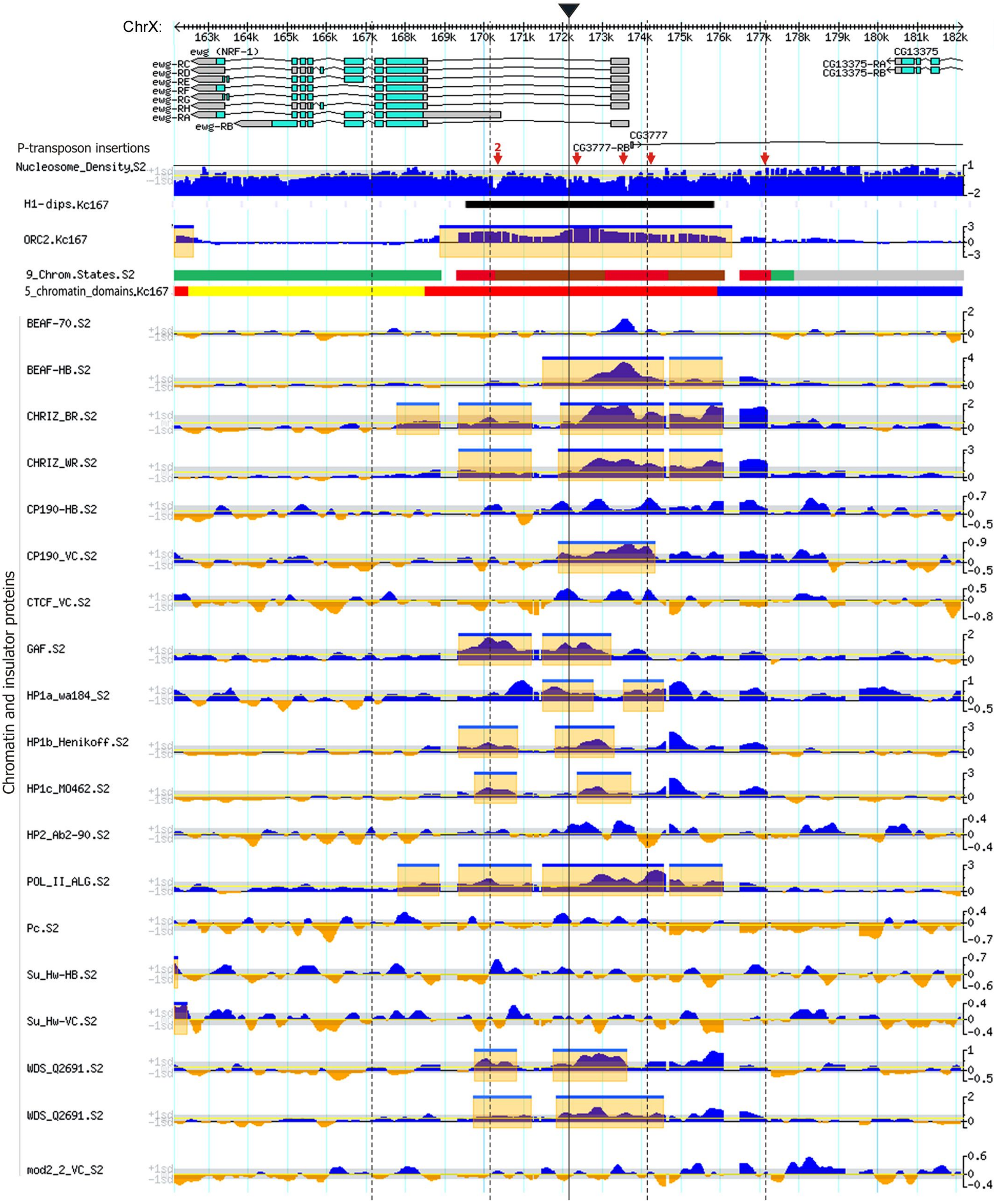

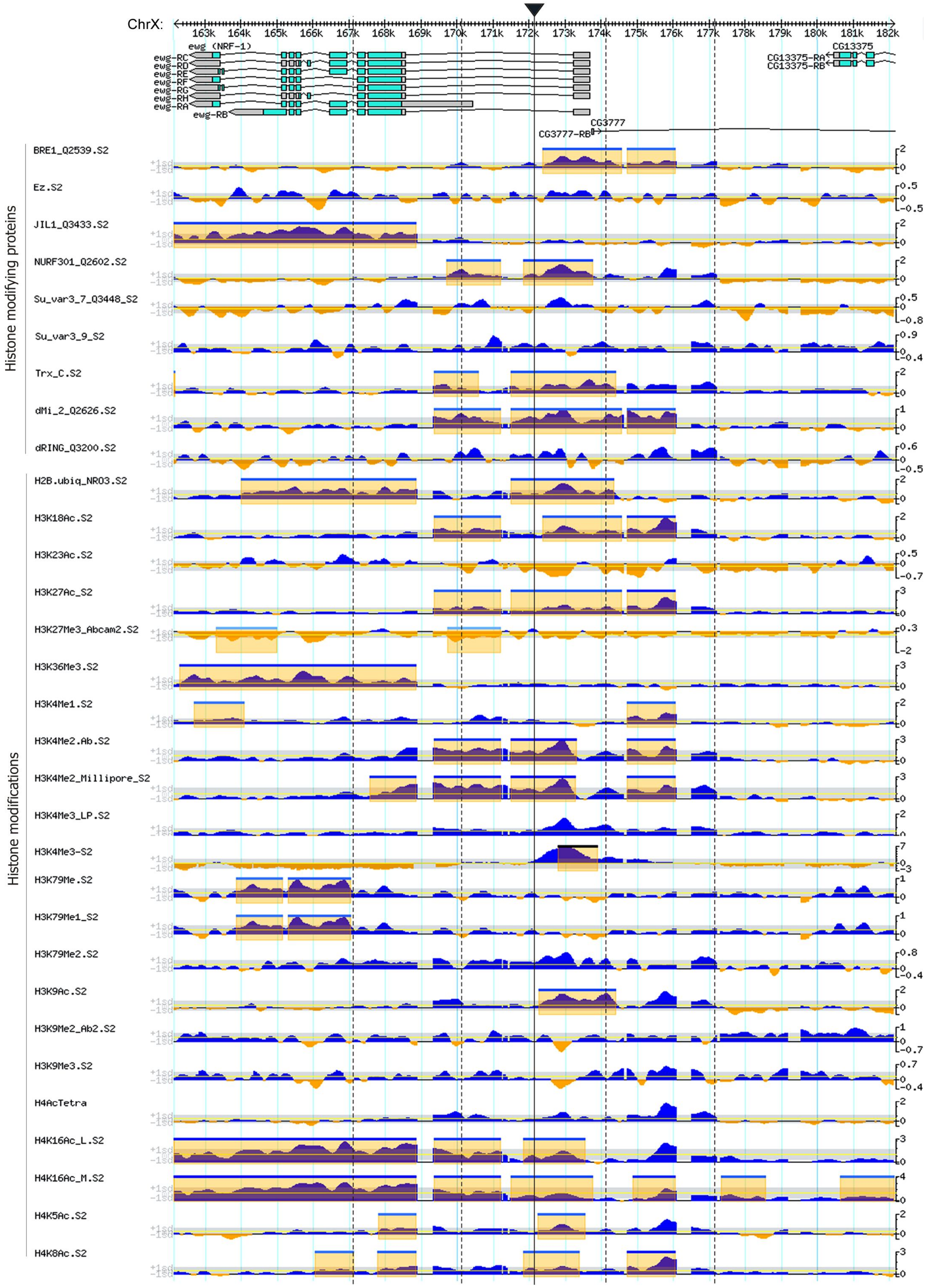

ChrX:
163k 164k 165k 166k 167k 168k 169k 170k 171k 172k 173k 174k 175k 176k 177k 178k 179k 180k 181k 182k
ewg (NRF-1)
ewg-RC
ewg-RD
ewg-RE
ewg-RF
ewg-RG
ewg-RH
ewg-RA
ewg-RB
CG13375
CG13375-RA
CG13375-RB
CG3777
CG3777-RB
Histone modifying proteins
BRE1_Q2539.S2
Ez.S2
JIL1_Q3433.S2
NURF301_Q2602.S2
Su_var3_7_Q3448_S2
Su_var3_9_S2
Trx_C.S2
dMi_2_Q2626.S2
dRING_Q3200.S2
Histone modifications
H2B.ubiq_NRO3.S2
H3K18Ac.S2
H3K23Ac.S2
H3K27Ac_S2
H3K27Me3_Abcam2.S2
H3K36Me3.S2
H3K4Me1.S2
H3K4Me2.Ab.S2
H3K4Me2_Millipore_S2
H3K4Me3_LP.S2
H3K4Me3-S2
H3K79Me.S2
H3K79Me1_S2
H3K79Me2.S2
H3K9Ac.S2
H3K9Me2_Ab2.S2
H3K9Me3.S2
H4AcTetra
H4K16Ac_L.S2
H4K16Ac_M.S2
H4K5Ac.S2
H4K8Ac.S2

Region 3A5/A6

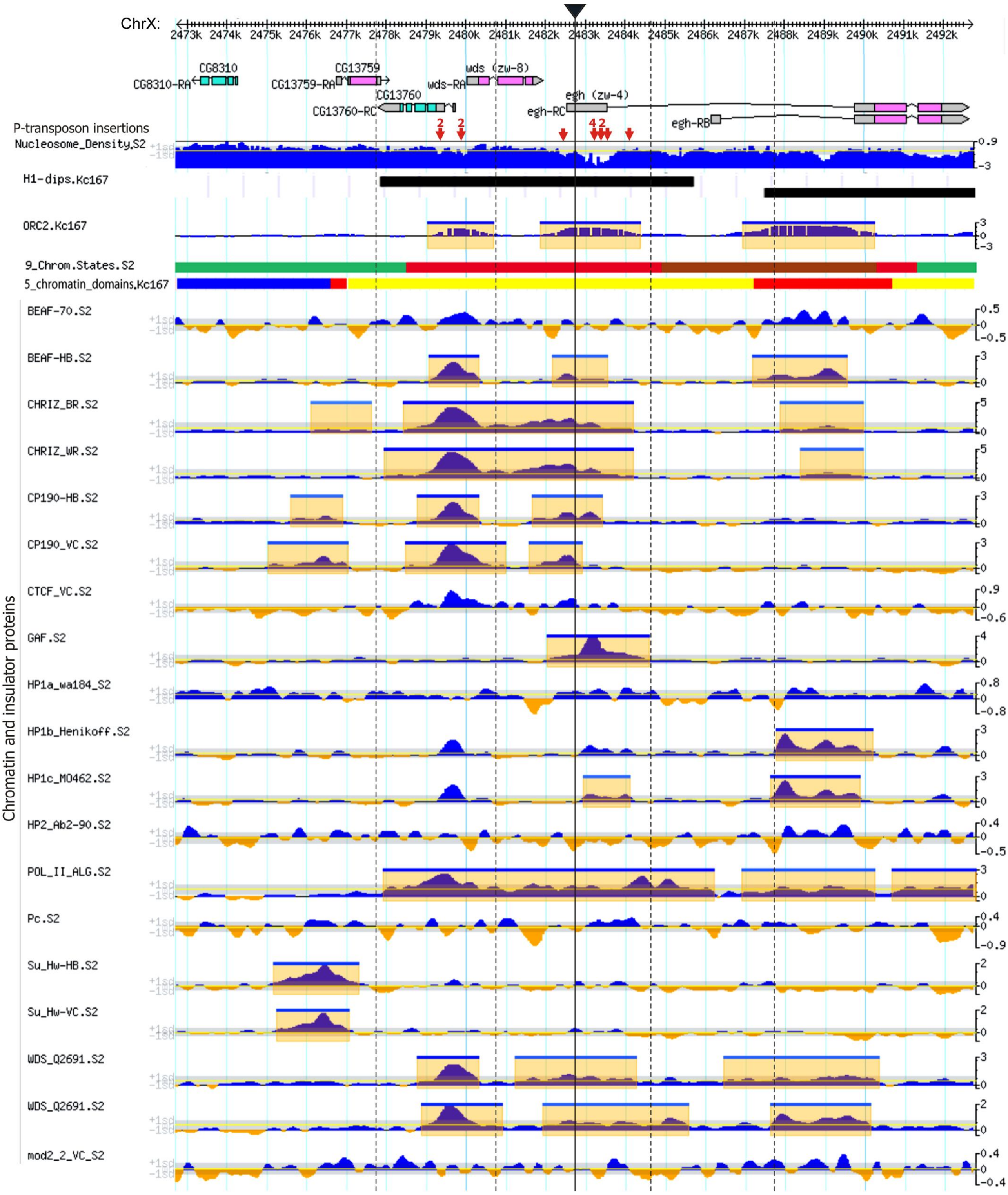

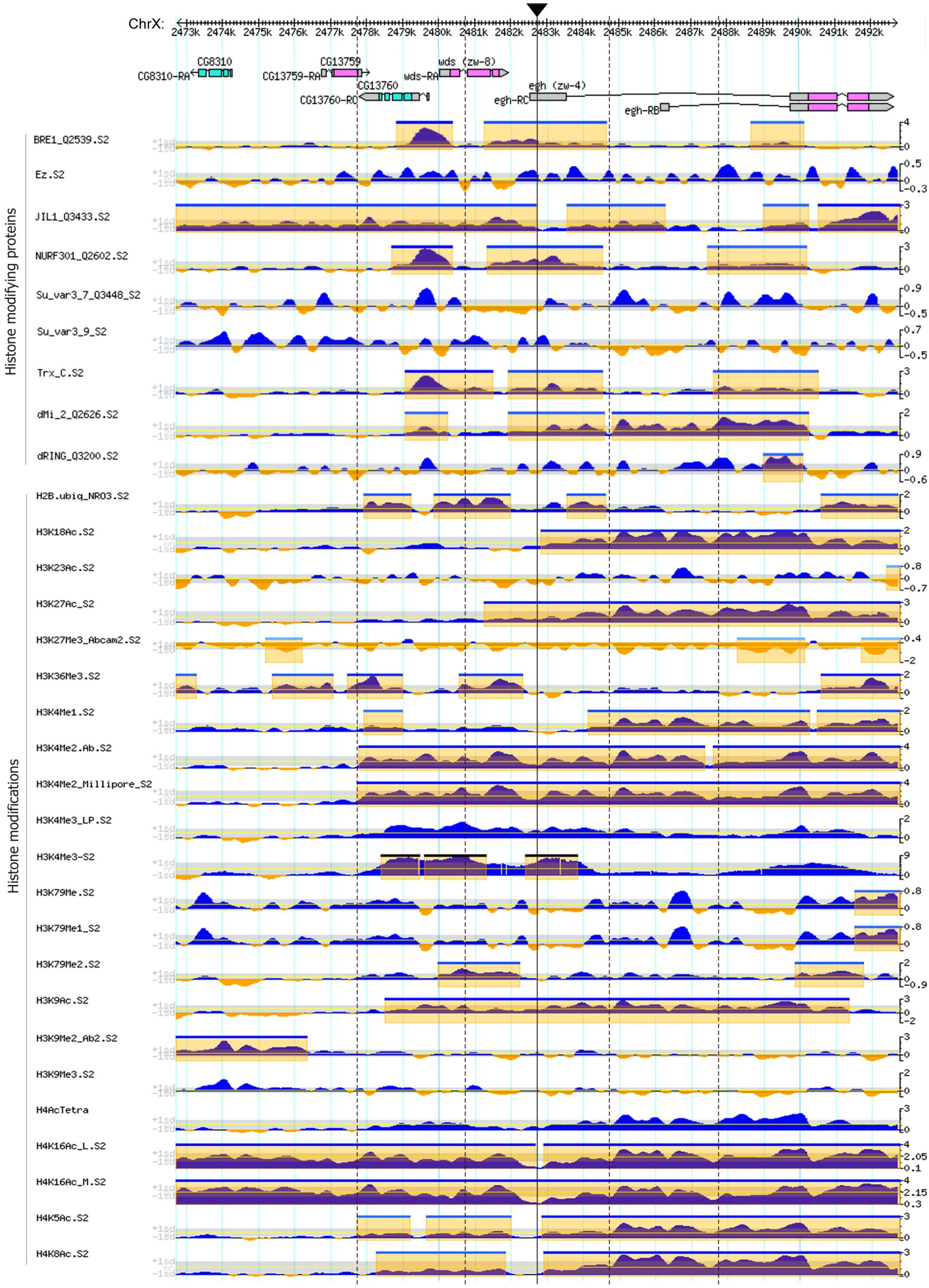

ChrX:
2473k 2474k 2475k 2476k 2477k 2478k 2479k 2480k 2481k 2482k 2483k 2484k 2485k 2486k 2487k 2488k 2489k 2490k 2491k 2492k
CG8310
CG8310-RA
CG13759
CG13759-RA
CG13760
CG13760-RC
wds (zw-8)
wds-RA
egh (zw-4)
egh-RC
egh-RB
Histone modifying proteins
BRE1_Q2539.S2
Ez.S2
JIL1_Q3433.S2
NURF301_Q2602.S2
Su_var3_7_Q3448_S2
Su_var3_9_S2
Trx_C.S2
dMi_2_Q2626.S2
dRING_Q3200.S2
Histone modifications
H2B.ubiq_NRO3.S2
H3K18Ac.S2
H3K23Ac.S2
H3K27Ac_S2
H3K27Me3_Abcam2.S2
H3K36Me3.S2
H3K4Me1.S2
H3K4Me2.Ab.S2
H3K4Me2_Millipore_S2
H3K4Me3_LP.S2
H3K4Me3-S2
H3K79Me.S2
H3K79Me1_S2
H3K79Me2.S2
H3K9Ac.S2
H3K9Me2_Ab2.S2
H3K9Me3.S2
H4AcTetra
H4K16Ac_L.S2
H4K16Ac_M.S2
H4K5Ac.S2
H4K8Ac.S2

Region 3C5-6/C7

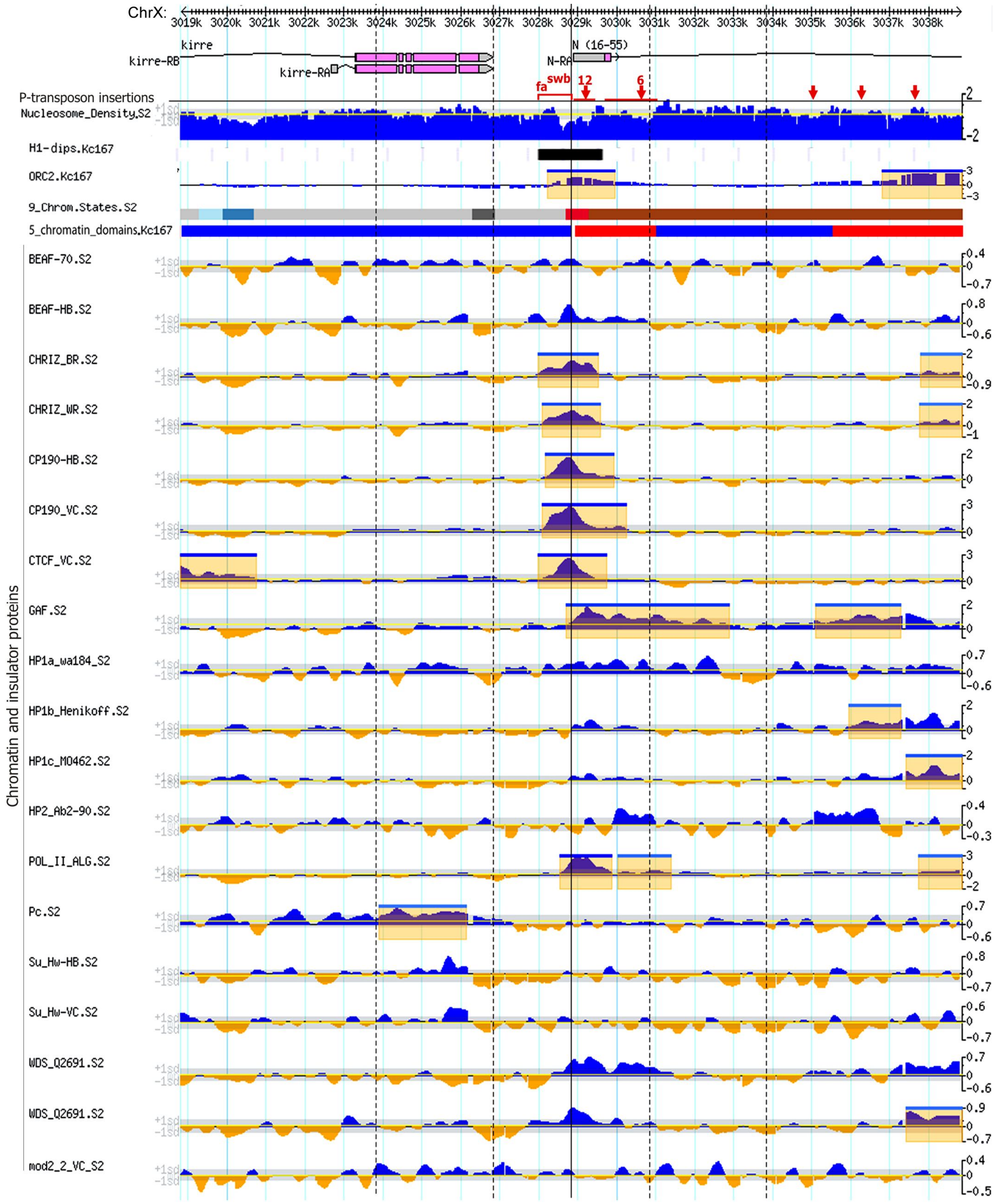

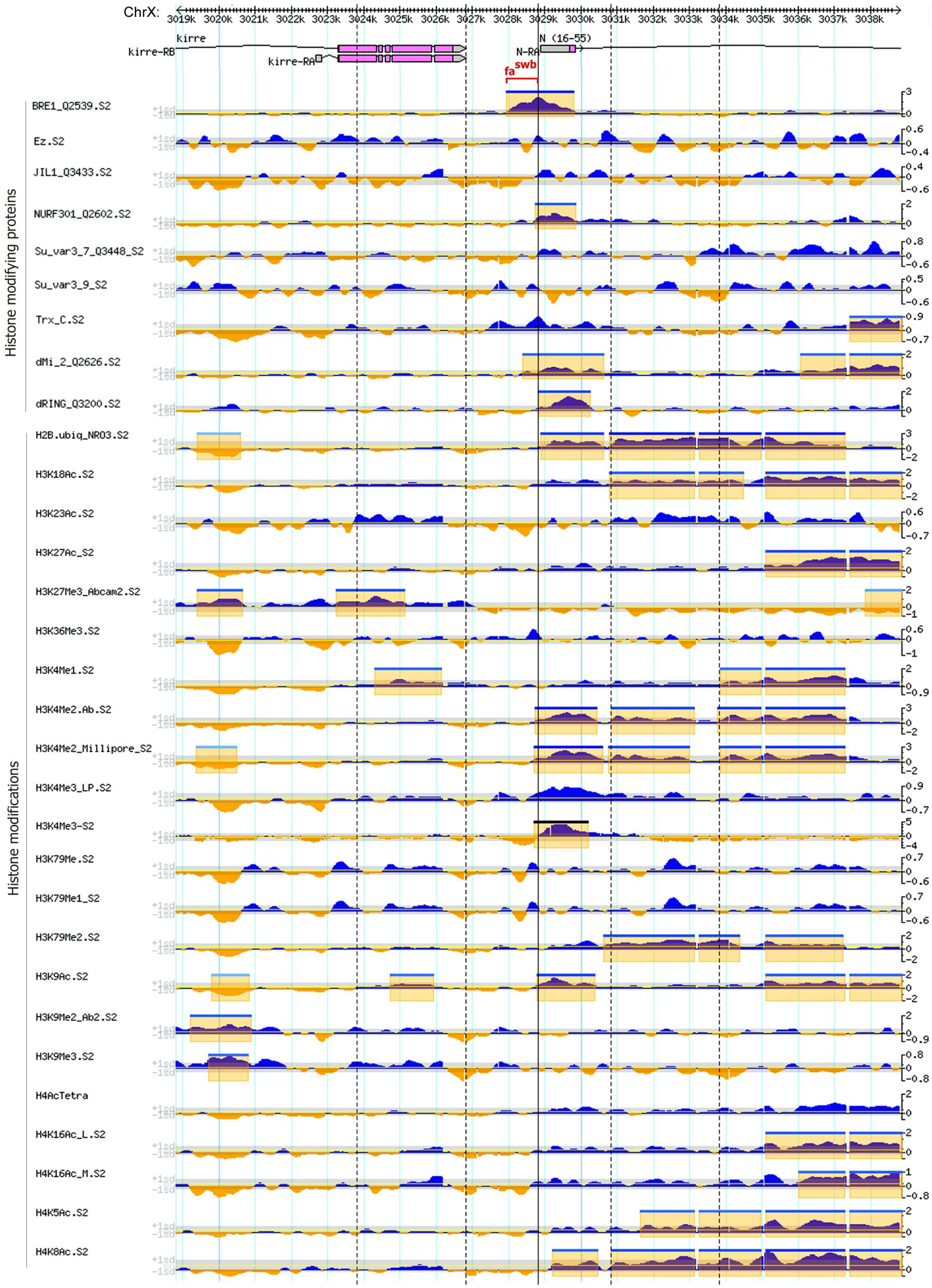
ChrX:
3019k 3020k 3021k 3022k 3023k 3024k 3025k 3026k 3027k 3028k 3029k 3030k 3031k 3032k 3033k 3034k 3035k 3036k 3037k 3038k
kirre
kirre-RB
kirre-RA
N (16-55)
N-RA
fa swb
Histone modifying proteins
BRE1_Q2539.S2
Ez.S2
JIL1_Q3433.S2
NURF301_Q2602.S2
Su_var3_7_Q3448_S2
Su_var3_9_S2
Trx_C.S2
dMi_2_Q2626.S2
dRING_Q3200.S2
Histone modifications
H2B.ubiq_NRO3.S2
H3K18Ac.S2
H3K23Ac.S2
H3K27Ac_S2
H3K27Me3_Abcam2.S2
H3K36Me3.S2
H3K4Me1.S2
H3K4Me2.Ab.S2
H3K4Me2_Millipore_S2
H3K4Me3_LP.S2
H3K4Me3-S2
H3K79Me.S2
H3K79Me1_S2
H3K79Me2.S2
H3K9Ac.S2
H3K9Me2_Ab2.S2
H3K9Me3.S2
H4AcTetra
H4K16Ac_L.S2
H4K16Ac_M.S2
H4K5Ac.S2
H4K8Ac.S2

Region 5F3/F5-6

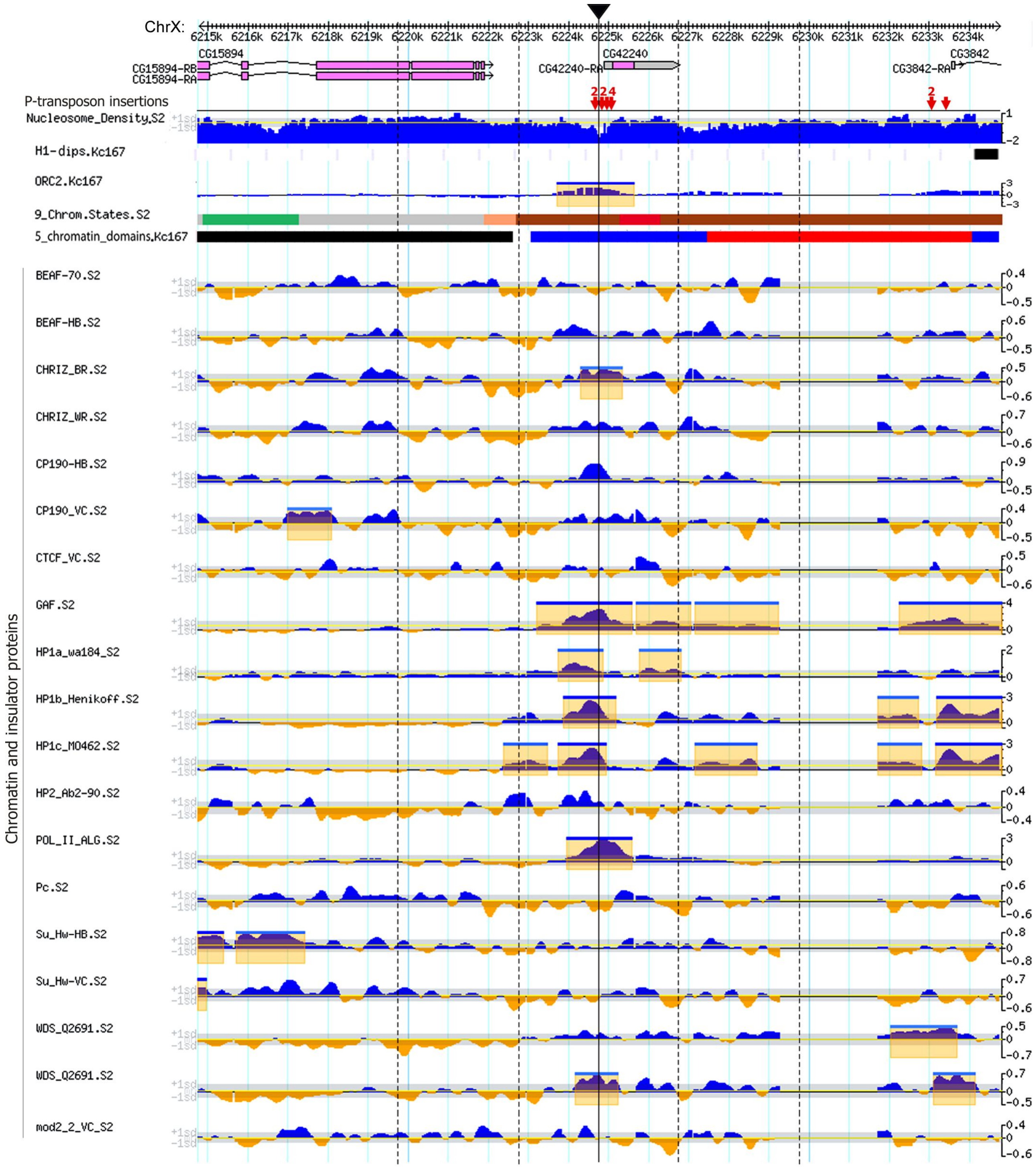

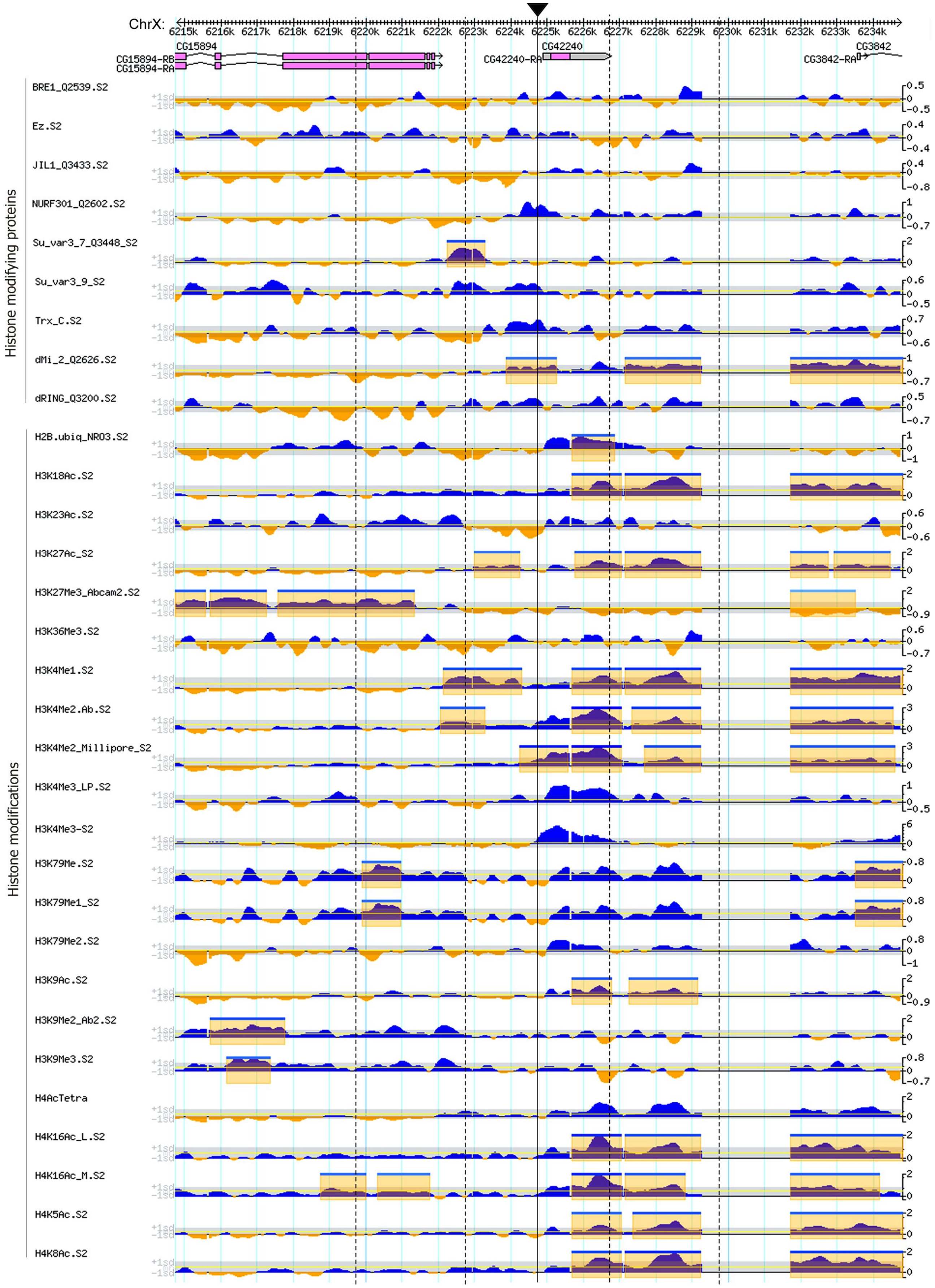

ChrX:
6215k 6216k 6217k 6218k 6219k 6220k 6221k 6222k 6223k 6224k 6225k 6226k 6227k 6228k 6229k 6230k 6231k 6232k 6233k 6234k
CG15894
CG15894-RB
CG15894-RA
CG42240
CG42240-RA
CG3842
CG3842-RA
Histone modifying proteins
BRE1_Q2539.S2
Ez.S2
JIL1_Q3433.S2
NURF301_Q2602.S2
Su_var3_7_Q3448_S2
Su_var3_9_S2
Trx_C.S2
dMi_2_Q2626.S2
dRING_Q3200.S2
Histone modifications
H2B.ubiq_NRO3.S2
H3K18Ac.S2
H3K23Ac.S2
H3K27Ac_S2
H3K27Me3_Abcam2.S2
H3K36Me3.S2
H3K4Me1.S2
H3K4Me2.Ab.S2
H3K4Me2_Millipore_S2
H3K4Me3_LP.S2
H3K4Me3-S2
H3K79Me.S2
H3K79Me1_S2
H3K79Me2.S2
H3K9Ac.S2
H3K9Me2_Ab2.S2
H3K9Me3.S2
H4AcTetra
H4K16Ac_L.S2
H4K16Ac_M.S2
H4K5Ac.S2
H4K8Ac.S2
+1sd
-1sd

Region 8E9/E10-11

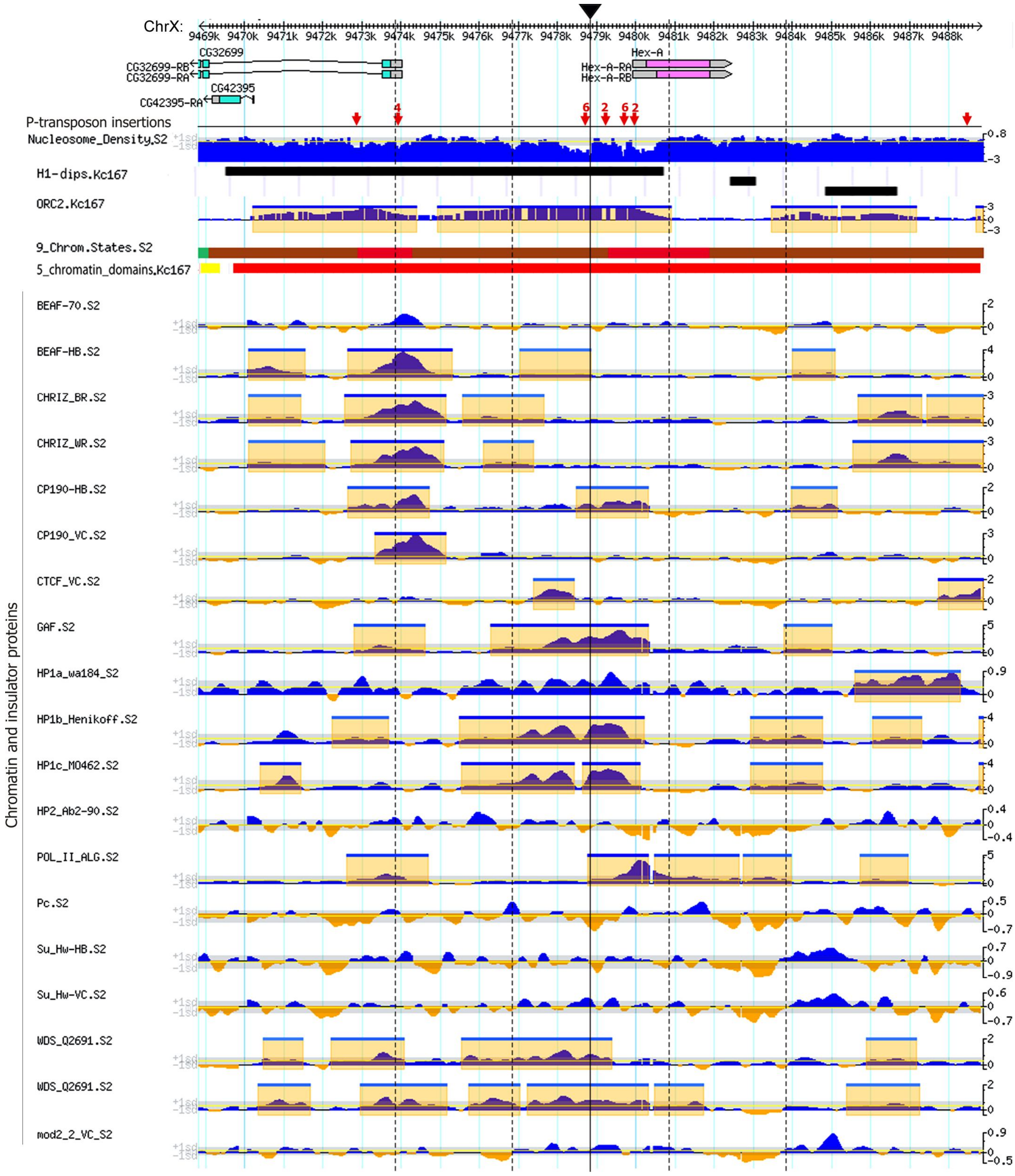

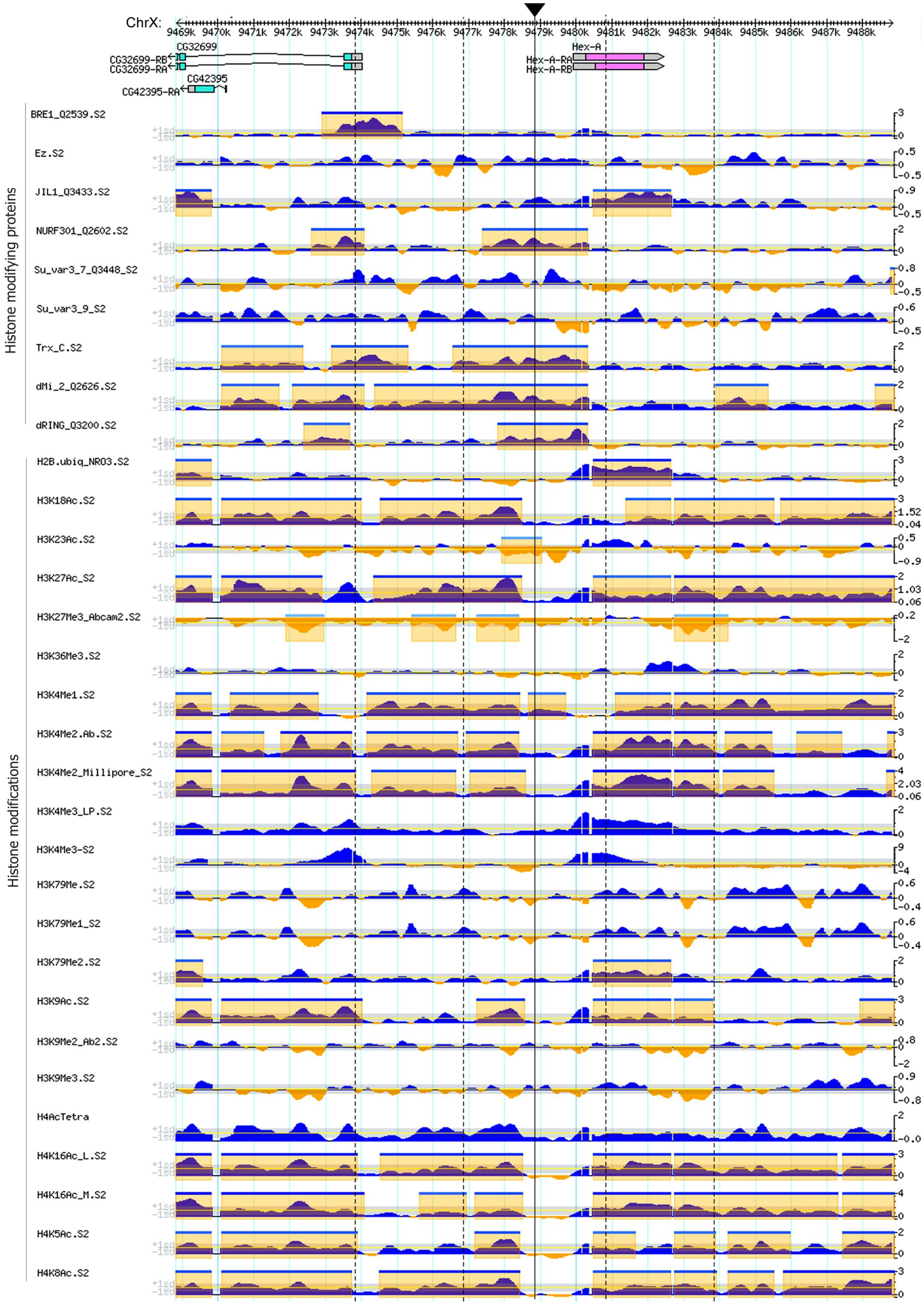
ChrX:
9469k 9470k 9471k 9472k 9473k 9474k 9475k 9476k 9477k 9478k 9479k 9480k 9481k 9482k 9483k 9484k 9485k 9486k 9487k 9488k
CG32699
CG32699-RB
CG32699-RA
CG42395
CG42395-RA
Hex-A
Hex-A-RA
Hex-A-RB
Histone modifying proteins
BRE1_Q2539.S2
Ez.S2
JIL1_Q3433.S2
NURF301_Q2602.S2
Su_var3_7_Q3448_S2
Su_var3_9_S2
Trx_C.S2
dMi_2_Q2626.S2
dRING_Q3200.S2
Histone modifications
H2B.ubiq_NRO3.S2
H3K18Ac.S2
H3K23Ac.S2
H3K27Ac_S2
H3K27Me3_Abcam2.S2
H3K36Me3.S2
H3K4Me1.S2
H3K4Me2.Ab.S2
H3K4Me2_Millipore_S2
H3K4Me3_LP.S2
H3K4Me3-S2
H3K79Me.S2
H3K79Me1_S2
H3K79Me2.S2
H3K9Ac.S2
H3K9Me2_Ab2.S2
H3K9Me3.S2
H4AcTetra
H4K16Ac_L.S2
H4K16Ac_M.S2
H4K5Ac.S2
H4K8Ac.S2
+1sd
-1sd

Region 60E8/E10

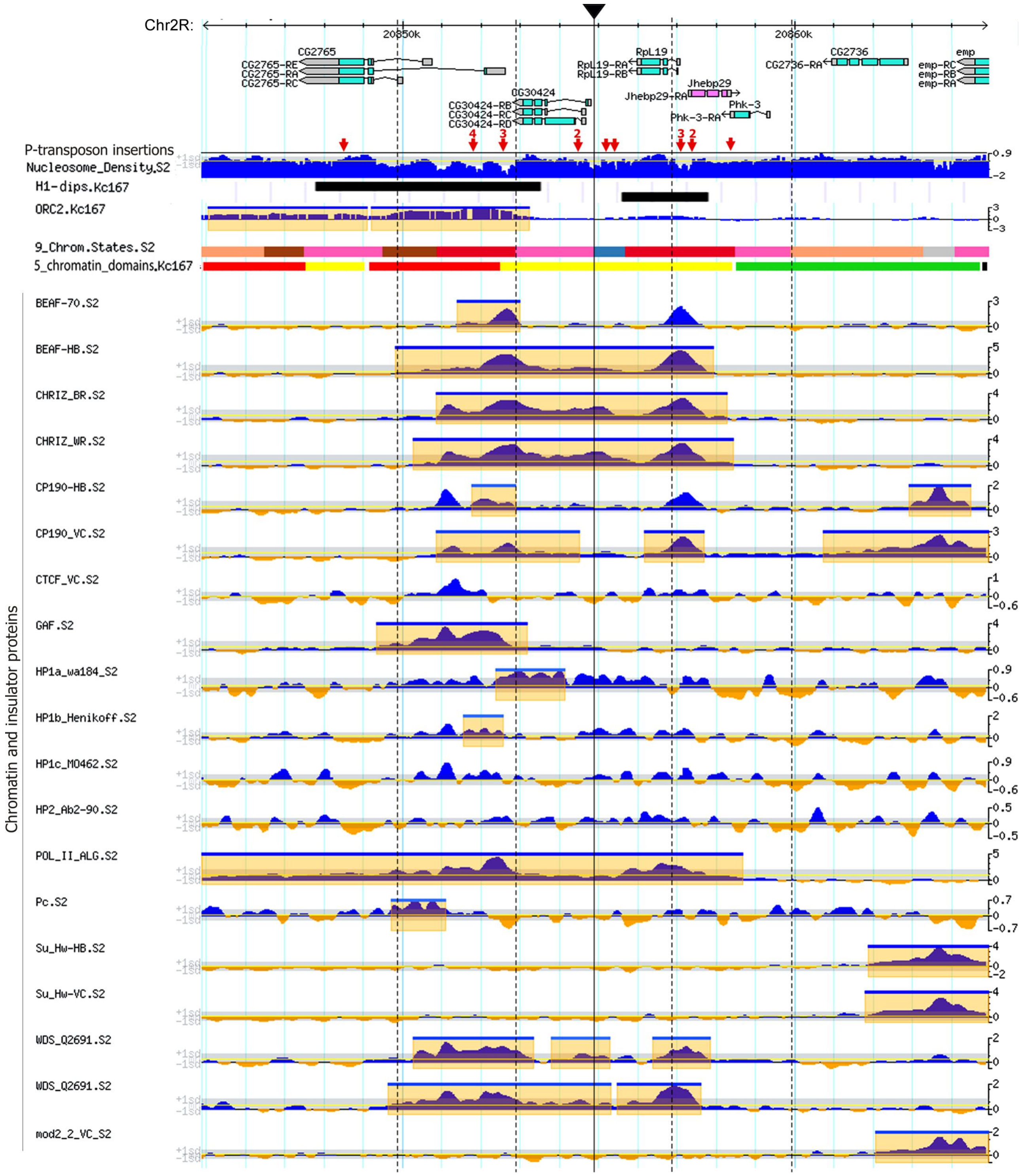

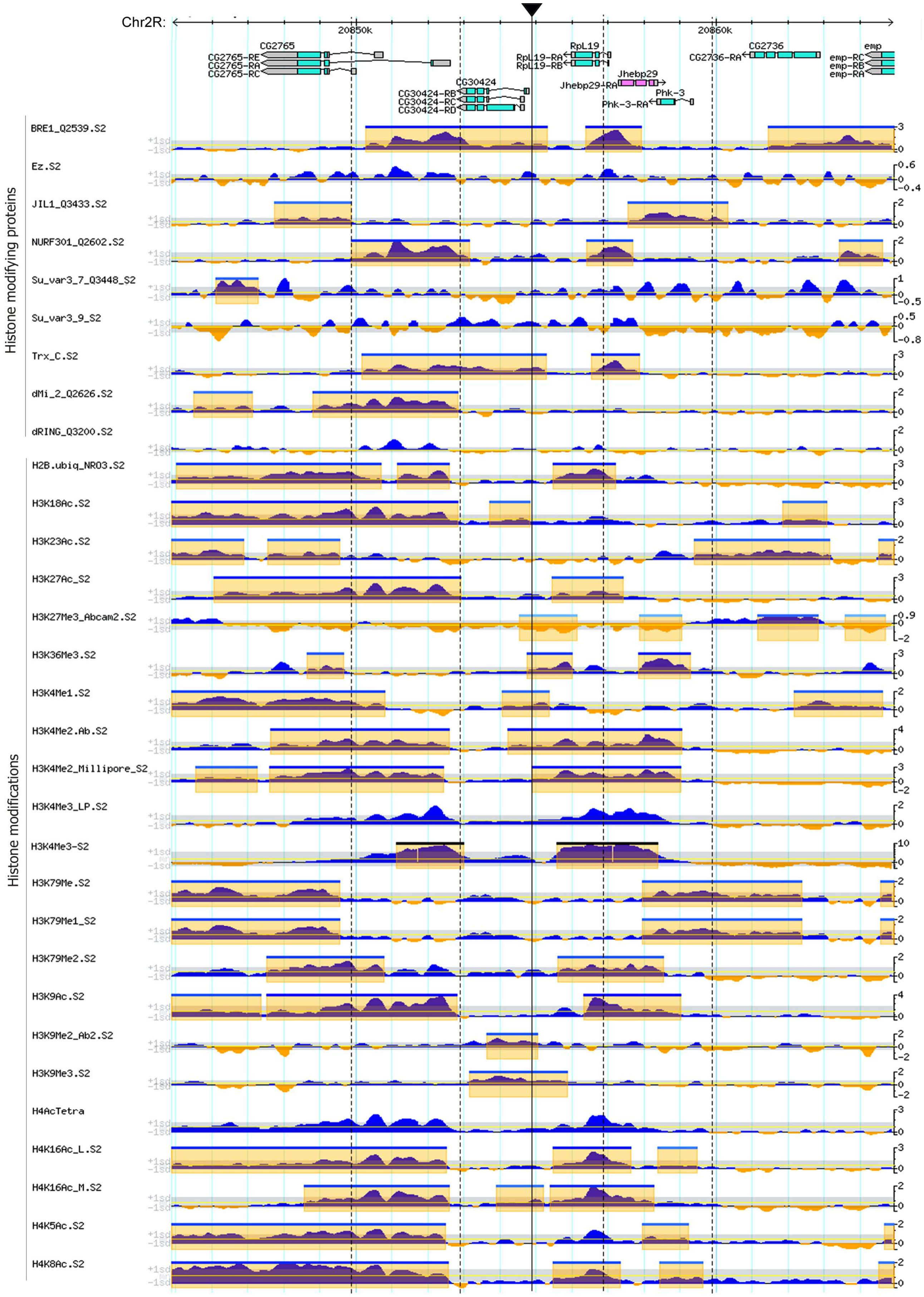
Chr2R:
20850k
20860k
CG2765
CG2765-RE
CG2765-RA
CG2765-RC
CG30424
CG30424-RB
CG30424-RC
CG30424-RD
RpL19
RpL19-RA
RpL19-RB
Jhebp29
Jhebp29-RA
Phk-3
Phk-3-RA
CG2736
CG2736-RA
emp
emp-RC
emp-RB
emp-RA
Histone modifying proteins
BRE1_Q2539.S2
Ez.S2
JIL1_Q3433.S2
NURF301_Q2602.S2
Su_var3_7_Q3448_S2
Su_var3_9_S2
Trx_C.S2
dMi_2_Q2626.S2
dRING_Q3200.S2
Histone modifications
H2B.ubiq_NRO3.S2
H3K18Ac.S2
H3K23Ac.S2
H3K27Ac_S2
H3K27Me3_Abcam2.S2
H3K36Me3.S2
H3K4Me1.S2
H3K4Me2.Ab.S2
H3K4Me2_Millipore_S2
H3K4Me3_LP.S2
H3K4Me3-S2
H3K79Me.S2
H3K79Me1_S2
H3K79Me2.S2
H3K9Ac.S2
H3K9Me2_Ab2.S2
H3K9Me3.S2
H4AcTetra
H4K16Ac_L.S2
H4K16Ac_M.S2
H4K5Ac.S2
H4K8Ac.S2

Region 61C7/C8

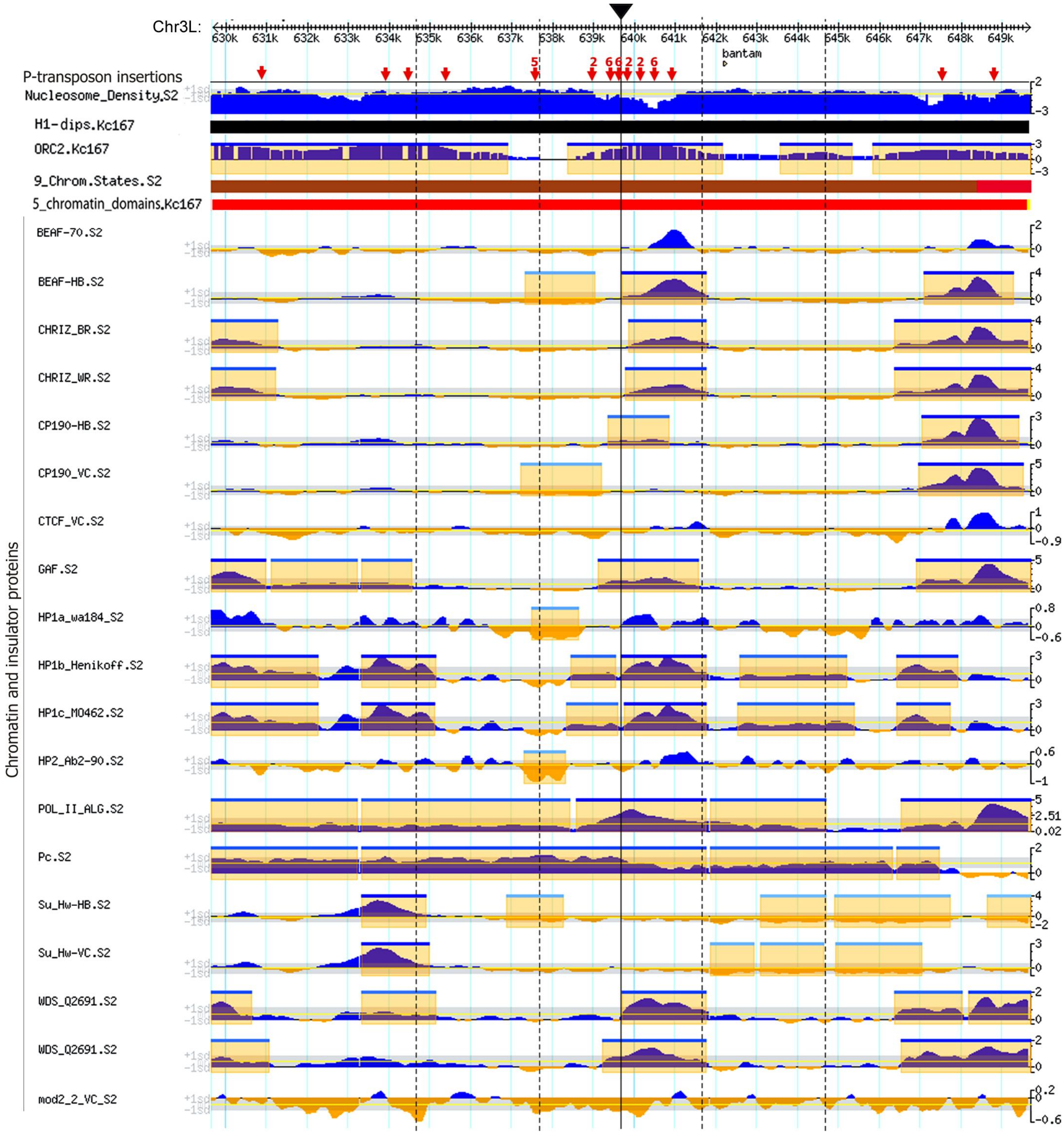

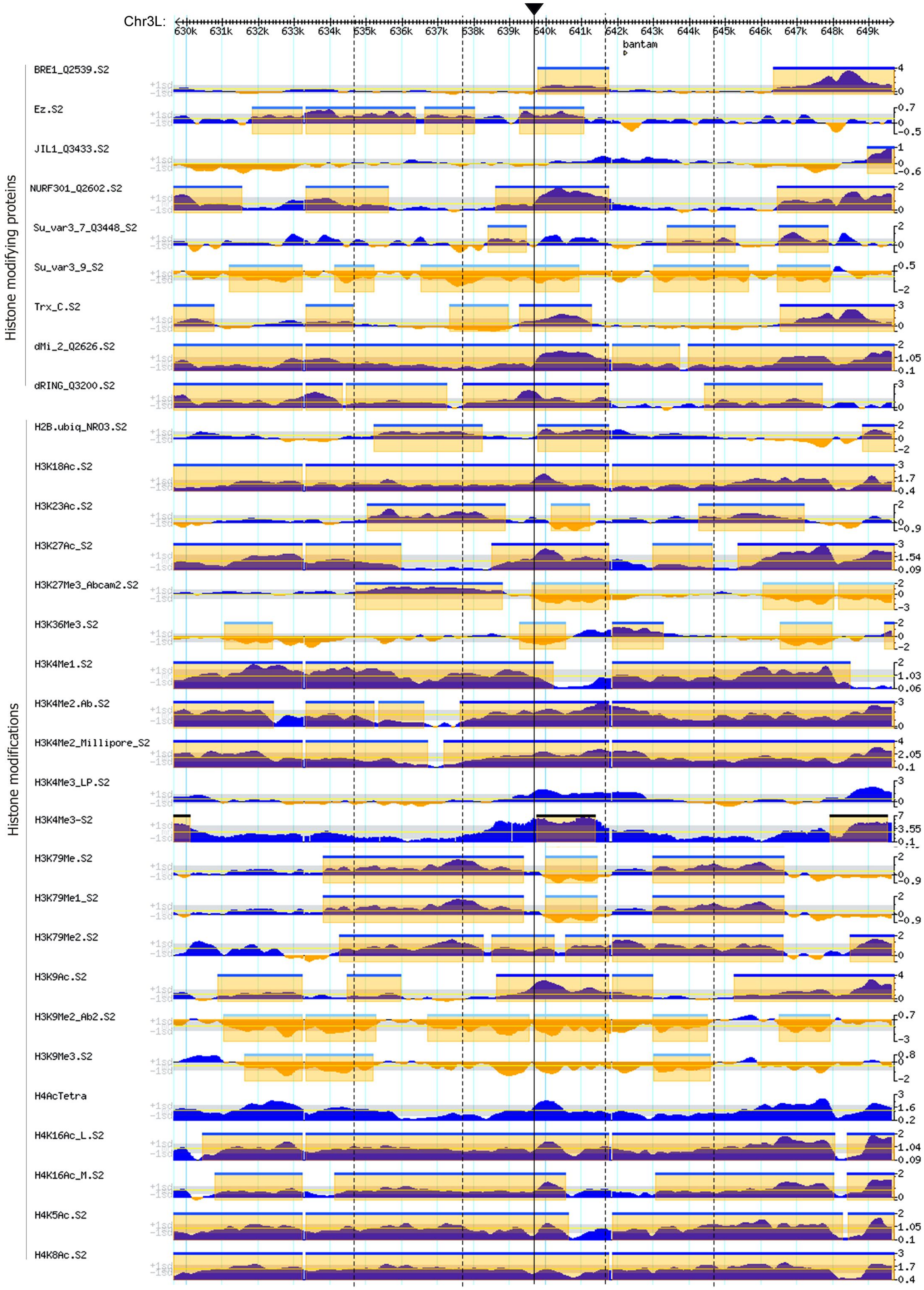

Chr3L:
630k 631k 632k 633k 634k 635k 636k 637k 638k 639k 640k 641k 642k 643k 644k 645k 646k 647k 648k 649k
bantam
Histone modifying proteins
BRE1_Q2539.S2
Ez.S2
JIL1_Q3433.S2
NURF301_Q2602.S2
Su_var3_7_Q3448_S2
Su_var3_9_S2
Trx_C.S2
dMi_2_Q2626.S2
dRING_Q3200.S2
Histone modifications
H2B.ubiq_NRO3.S2
H3K18Ac.S2
H3K23Ac.S2
H3K27Ac_S2
H3K27Me3_Abcam2.S2
H3K36Me3.S2
H3K4Me1.S2
H3K4Me2.Ab.S2
H3K4Me2_Millipore_S2
H3K4Me3_LP.S2
H3K4Me3-S2
H3K79Me.S2
H3K79Me1_S2
H3K79Me2.S2
H3K9Ac.S2
H3K9Me2_Ab2.S2
H3K9Me3.S2
H4AcTetra
H4K16Ac_L.S2
H4K16Ac_M.S2
H4K5Ac.S2
H4K8Ac.S2
+1sd
-1sd

Region 67B11/C1-2

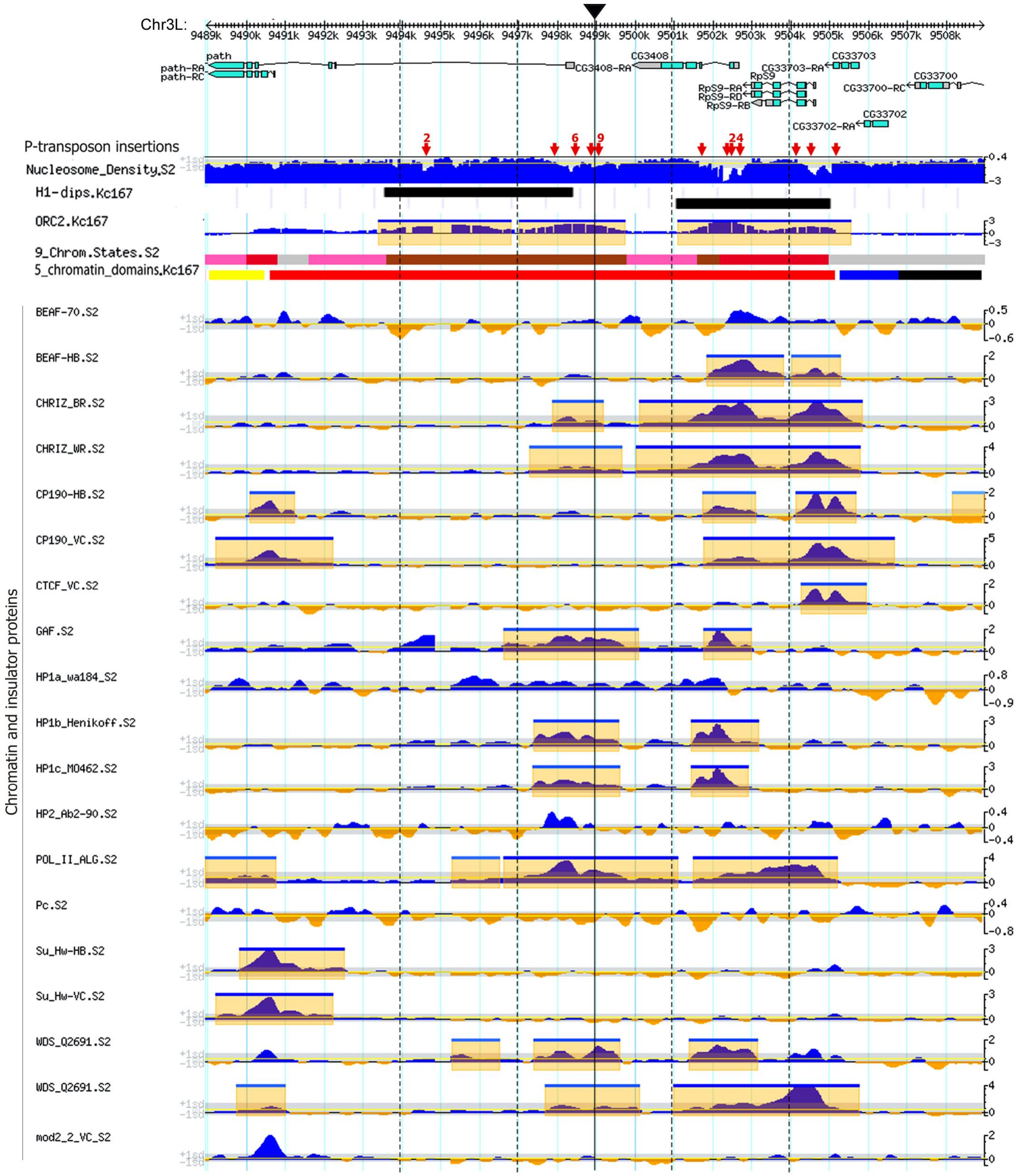

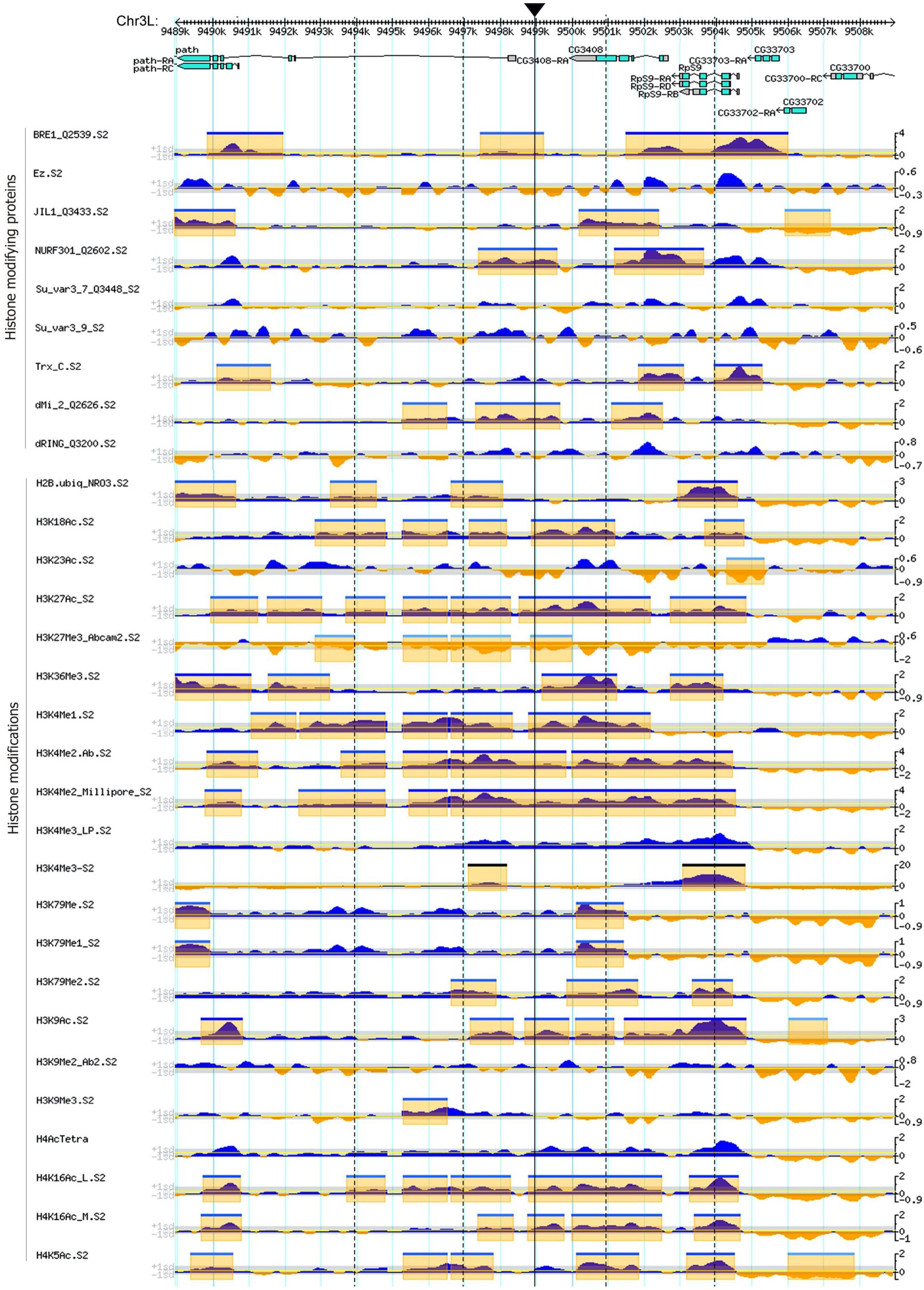
Chr3L:
9489k 9490k 9491k 9492k 9493k 9494k 9495k 9496k 9497k 9498k 9499k 9500k 9501k 9502k 9503k 9504k 9505k 9506k 9507k 9508k
path
path-RA
path-RC
CG3408
CG3408-RA
CG33703
CG33703-RA
RpS9
RpS9-RA
RpS9-RD
RpS9-RB
CG33700
CG33700-RC
CG33702
CG33702-RA
Histone modifying proteins
BRE1_Q2539.S2
Ez.S2
JIL1_Q3433.S2
NURF301_Q2602.S2
Su_var3_7_Q3448_S2
Su_var3_9_S2
Trx_C.S2
dMi_2_Q2626.S2
dRING_Q3200.S2
Histone modifications
H2B.ubiq_NRO3.S2
H3K18Ac.S2
H3K23Ac.S2
H3K27Ac_S2
H3K27Me3_Abcam2.S2
H3K36Me3.S2
H3K4Me1.S2
H3K4Me2.Ab.S2
H3K4Me2_Millipore_S2
H3K4Me3_LP.S2
H3K4Me3-S2
H3K79Me.S2
H3K79Me1_S2
H3K79Me2.S2
H3K9Ac.S2
H3K9Me2_Ab2.S2
H3K9Me3.S2
H4AcTetra
H4K16Ac_L.S2
H4K16Ac_M.S2
H4K5Ac.S2
+1sd
-1sd

Region 79D2/D3

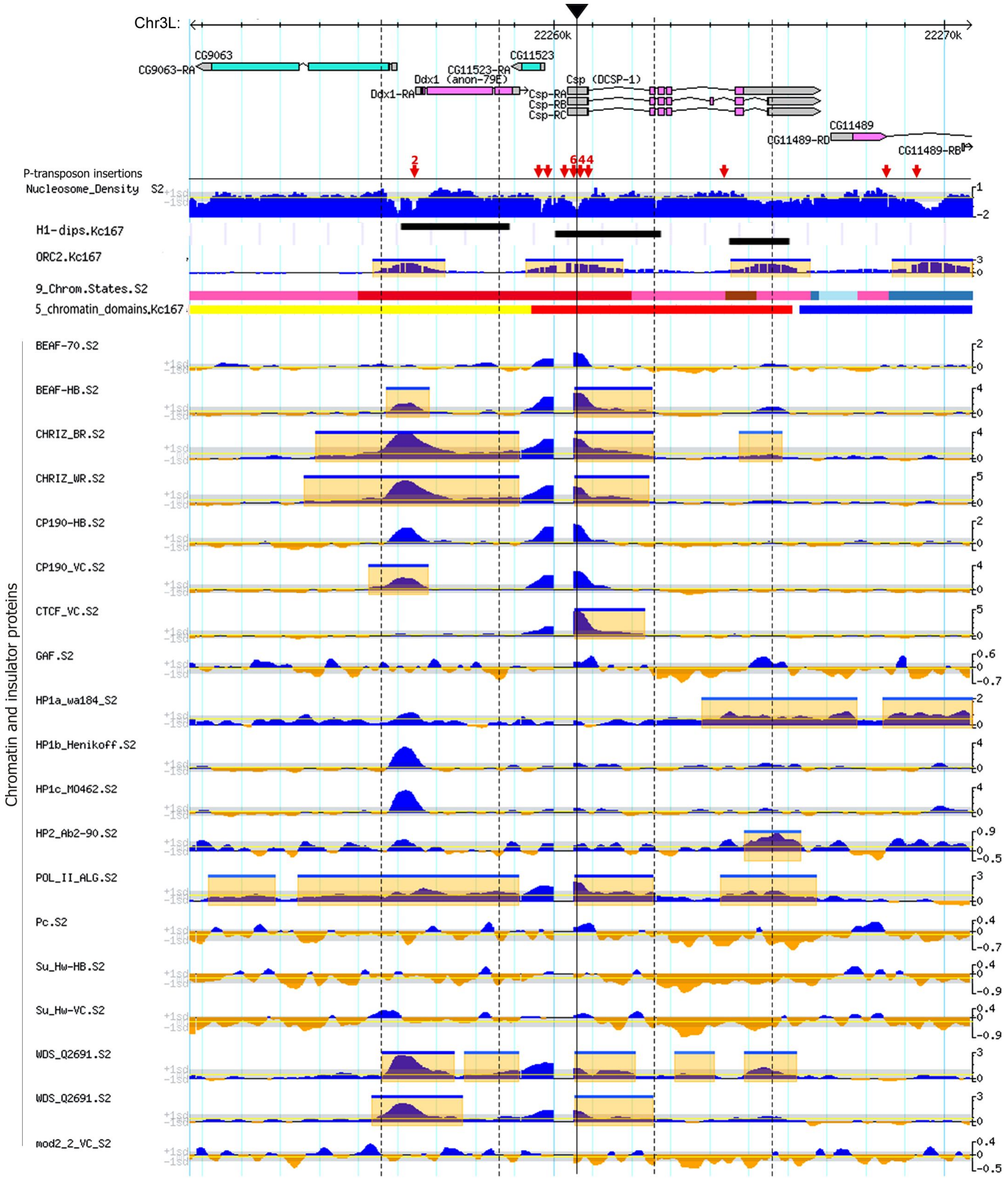

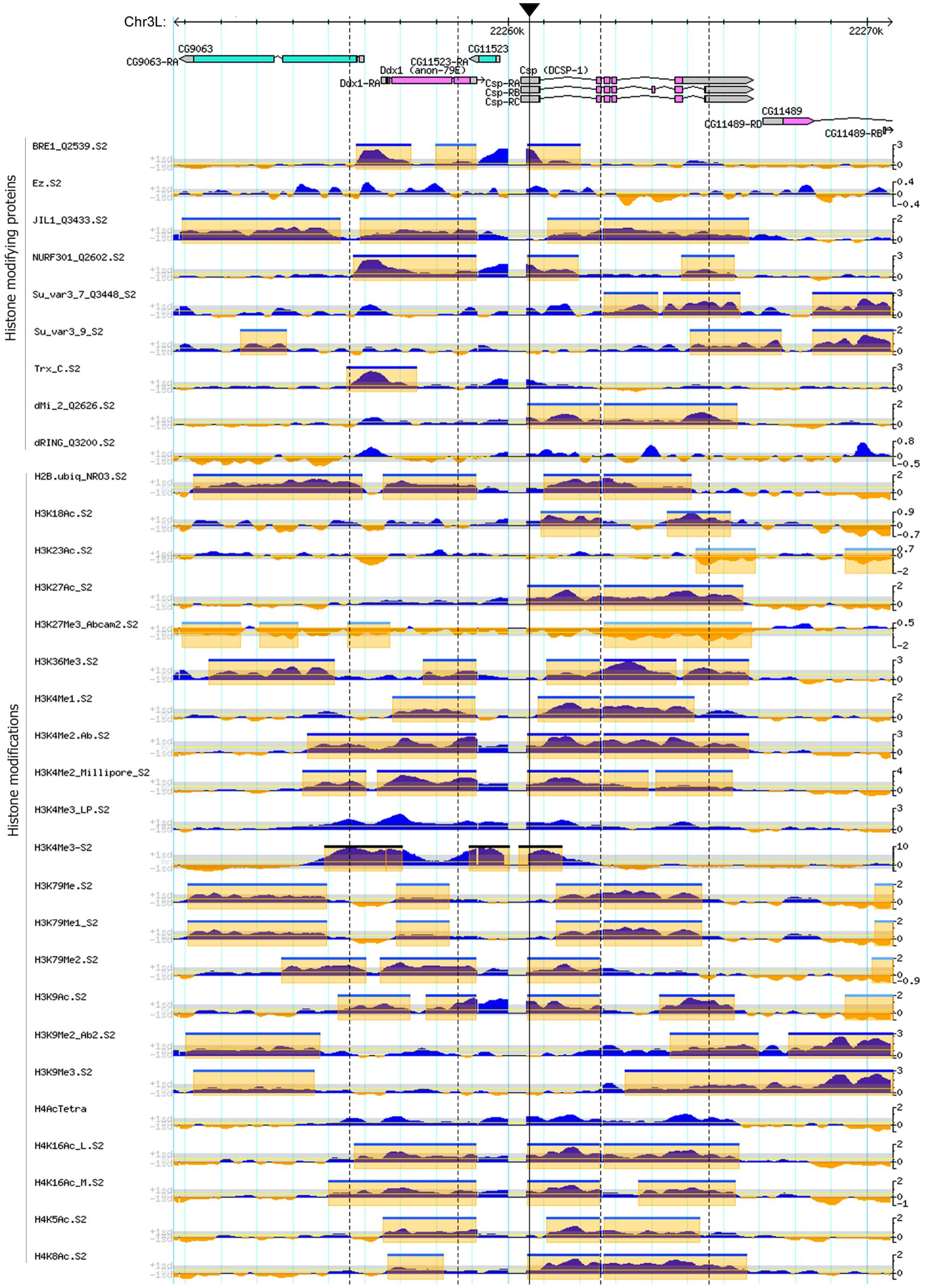
Chr3L:
22260k
22270k
CG9063
CG9063-RA
CG11523
CG11523-RA
Ddx1 (anon-79E)
Ddx1-RA
Csp (DCSP-1)
Csp-RA
Csp-RB
Csp-RC
CG11489
CG11489-RD
CG11489-RB
Histone modifying proteins
BRE1_Q2539.S2
Ez.S2
JIL1_Q3433.S2
NURF301_Q2602.S2
Su_var3_7_Q3448_S2
Su_var3_9_S2
Trx_C.S2
dMi_2_Q2626.S2
dRING_Q3200.S2
Histone modifications
H2B.ubiq_NRO3.S2
H3K18Ac.S2
H3K23Ac.S2
H3K27Ac_S2
H3K27Me3_Abcam2.S2
H3K36Me3.S2
H3K4Me1.S2
H3K4Me2.Ab.S2
H3K4Me2_Millipore_S2
H3K4Me3_LP.S2
H3K4Me3-S2
H3K79Me.S2
H3K79Me1_S2
H3K79Me2.S2
H3K9Ac.S2
H3K9Me2_Ab2.S2
H3K9Me3.S2
H4AcTetra
H4K16Ac_L.S2
H4K16Ac_M.S2
H4K5Ac.S2
H4K8Ac.S2

Region 84F10/F11-12

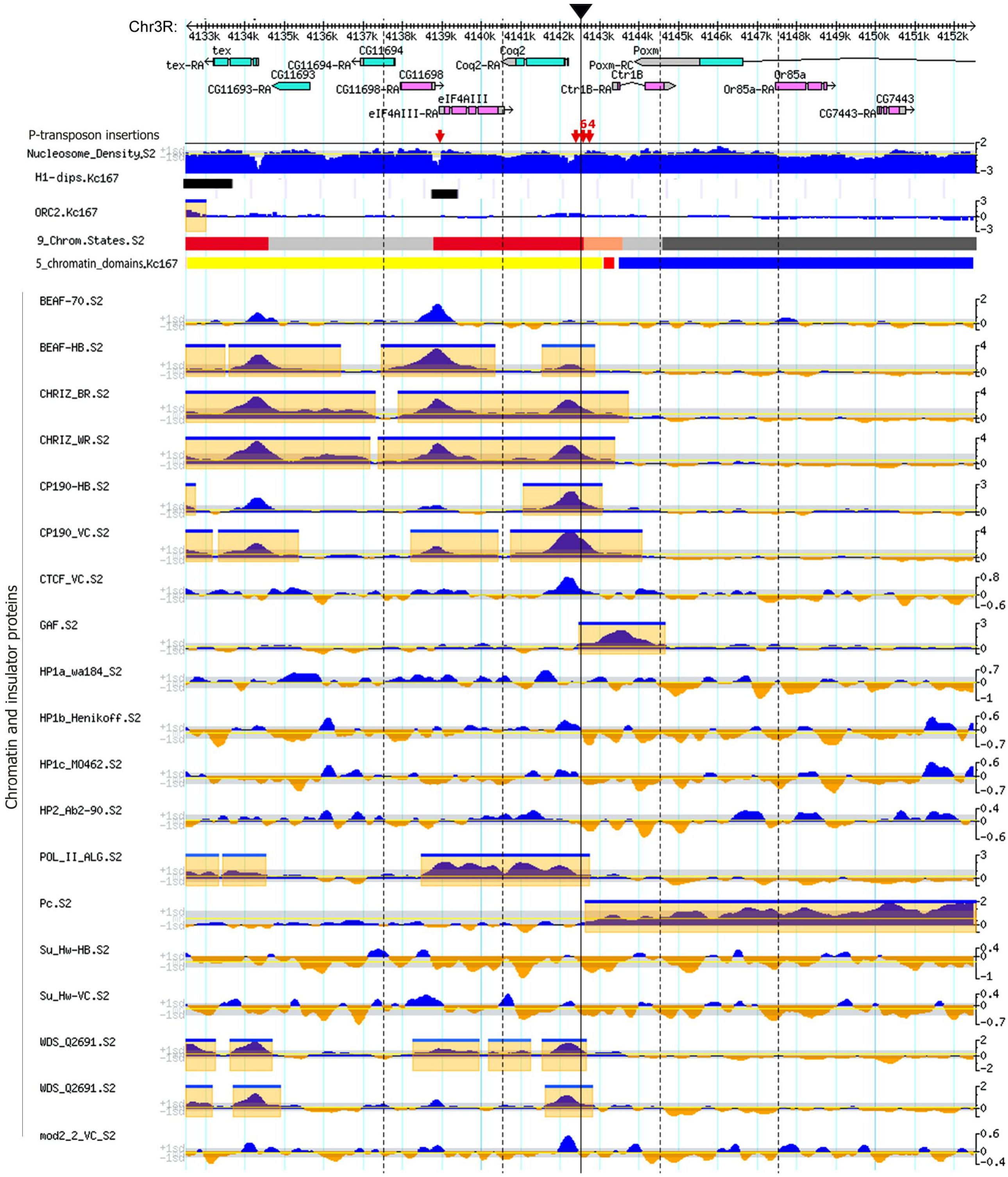

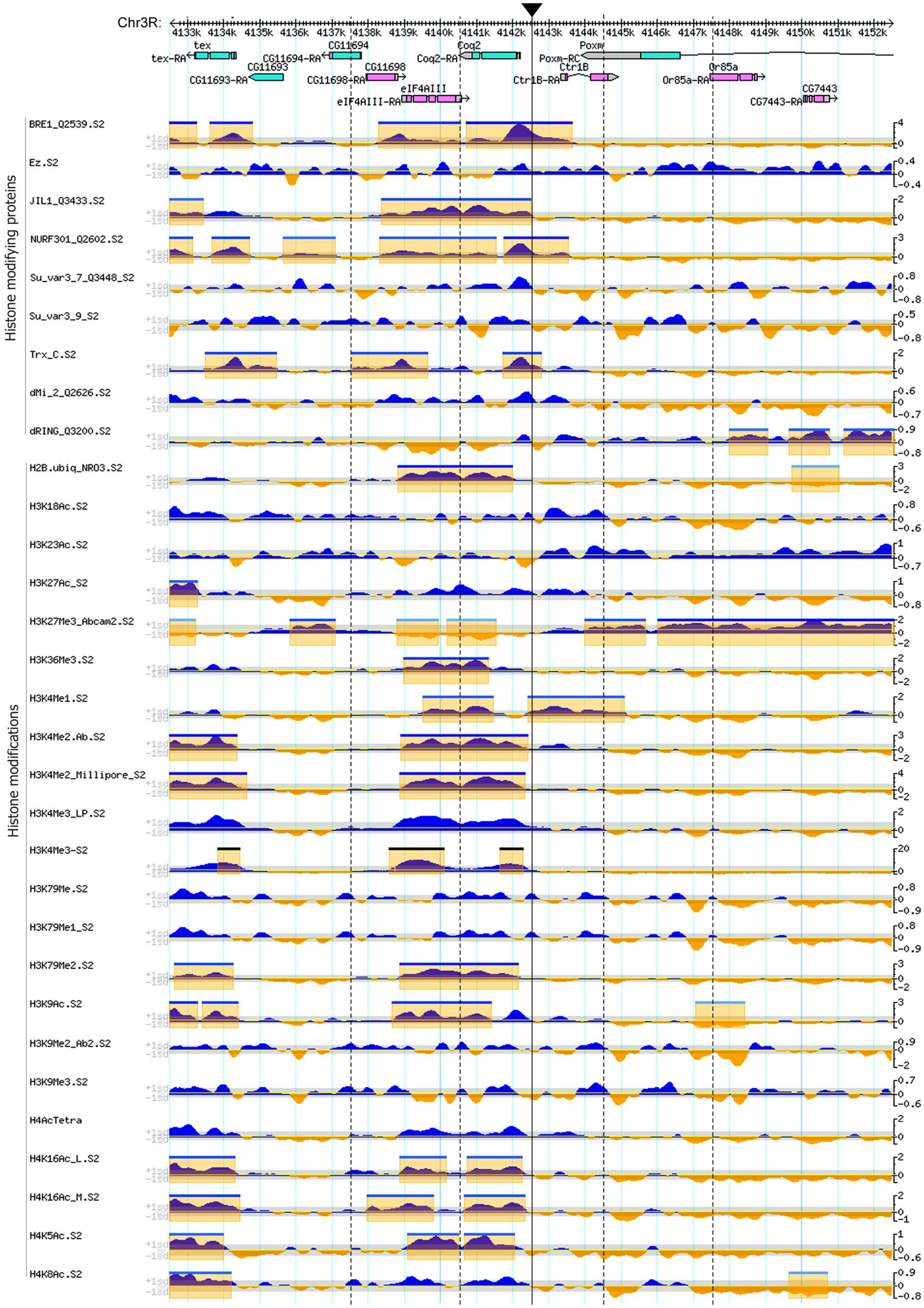

Chr3R:
4133k 4134k 4135k 4136k 4137k 4138k 4139k 4140k 4141k 4142k 4143k 4144k 4145k 4146k 4147k 4148k 4149k 4150k 4151k 4152k
tex
tex-RA
CG11694
CG11694-RA
CG11693
CG11693-RA
CG11698
CG11698-RA
eIF4AIII
eIF4AIII-RA
Coq2
Coq2-RA
Poxm
Poxm-RC
Ctr1B
Ctr1B-RA
Or85a
Or85a-RA
CG7443
CG7443-RA
Histone modifying proteins
BRE1_Q2539.S2
Ez.S2
JIL1_Q3433.S2
NURF301_Q2602.S2
Su_var3_7_Q3448_S2
Su_var3_9_S2
Trx_C.S2
dMi_2_Q2626.S2
dRING_Q3200.S2
Histone modifications
H2B.ubiq_NRO3.S2
H3K18Ac.S2
H3K23Ac.S2
H3K27Ac_S2
H3K27Me3_Abcam2.S2
H3K36Me3.S2
H3K4Me1.S2
H3K4Me2.Ab.S2
H3K4Me2_Millipore_S2
H3K4Me3_LP.S2
H3K4Me3-S2
H3K79Me.S2
H3K79Me1_S2
H3K79Me2.S2
H3K9Ac.S2
H3K9Me2_Ab2.S2
H3K9Me3.S2
H4AcTetra
H4K16Ac_L.S2
H4K16Ac_M.S2
H4K5Ac.S2
H4K8Ac.S2
+1sd
-1sd

Region 85D9/D10

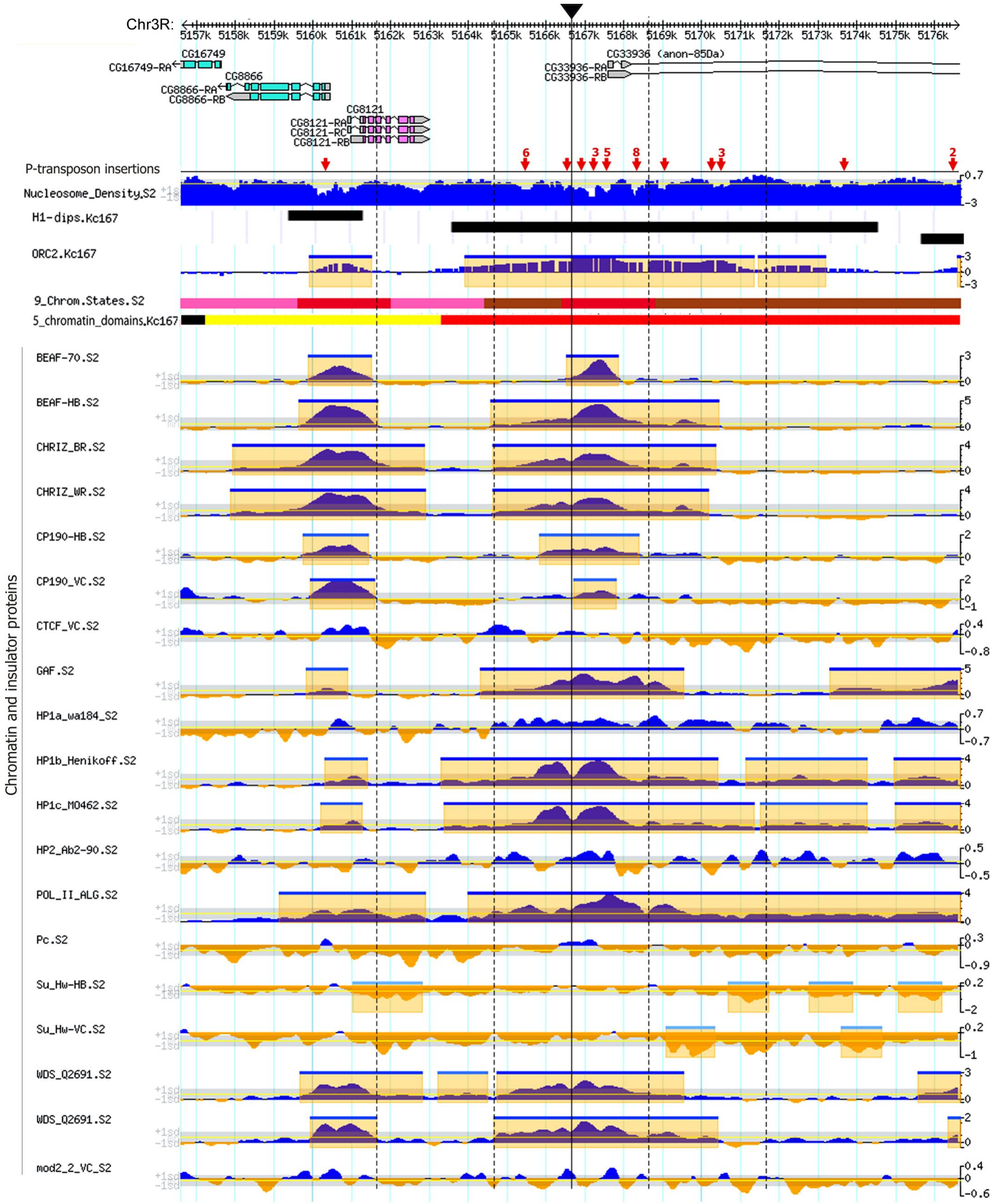

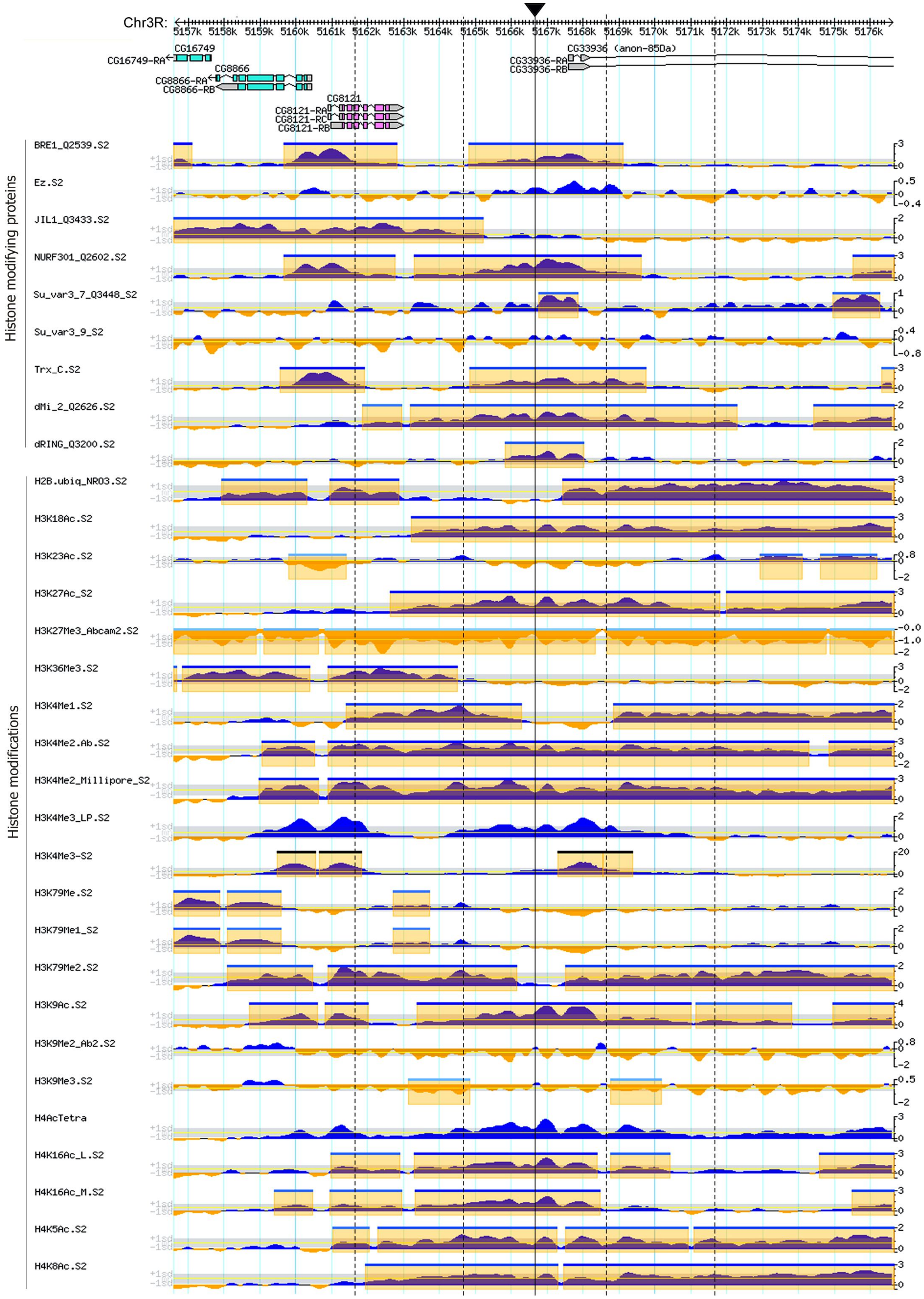
Chr3R:
5157k 5158k 5159k 5160k 5161k 5162k 5163k 5164k 5165k 5166k 5167k 5168k 5169k 5170k 5171k 5172k 5173k 5174k 5175k 5176k
CG16749
CG16749-RA
CG8866
CG8866-RA
CG8866-RB
CG8121
CG8121-RA
CG8121-RC
CG8121-RB
CG33936 (anon-85Da)
CG33936-RA
CG33936-RB
Histone modifying proteins
BRE1_Q2539.S2
Ez.S2
JIL1_Q3433.S2
NURF301_Q2602.S2
Su_var3_7_Q3448_S2
Su_var3_9_S2
Trx_C.S2
dMi_2_Q2626.S2
dRING_Q3200.S2
Histone modifications
H2B.ubiq_NRO3.S2
H3K18Ac.S2
H3K23Ac.S2
H3K27Ac_S2
H3K27Me3_Abcam2.S2
H3K36Me3.S2
H3K4Me1.S2
H3K4Me2.Ab.S2
H3K4Me2_Millipore_S2
H3K4Me3_LP.S2
H3K4Me3-S2
H3K79Me.S2
H3K79Me1_S2
H3K79Me2.S2
H3K9Ac.S2
H3K9Me2_Ab2.S2
H3K9Me3.S2
H4AcTetra
H4K16Ac_L.S2
H4K16Ac_M.S2
H4K5Ac.S2
H4K8Ac.S2

Region 86B4/B6

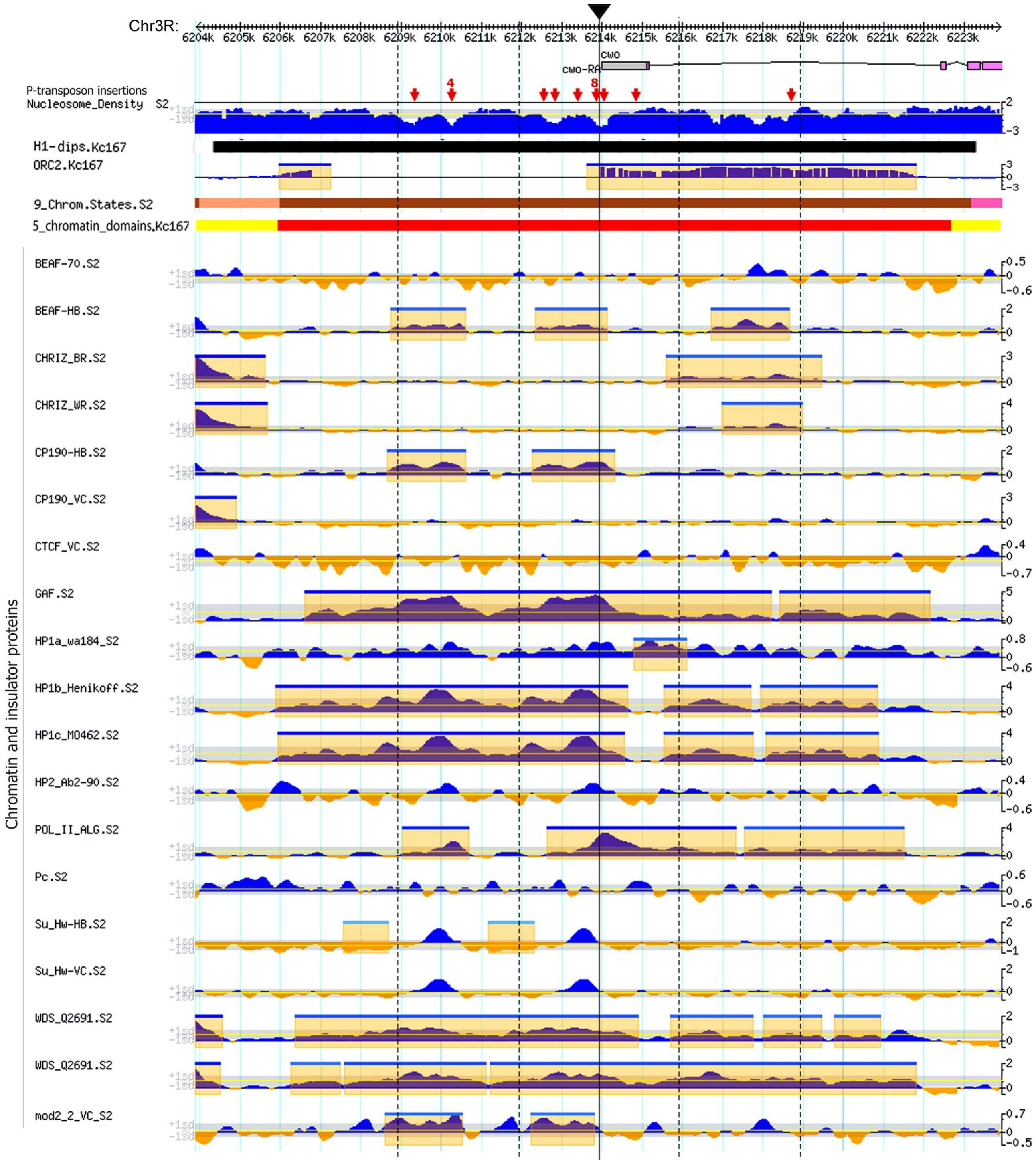

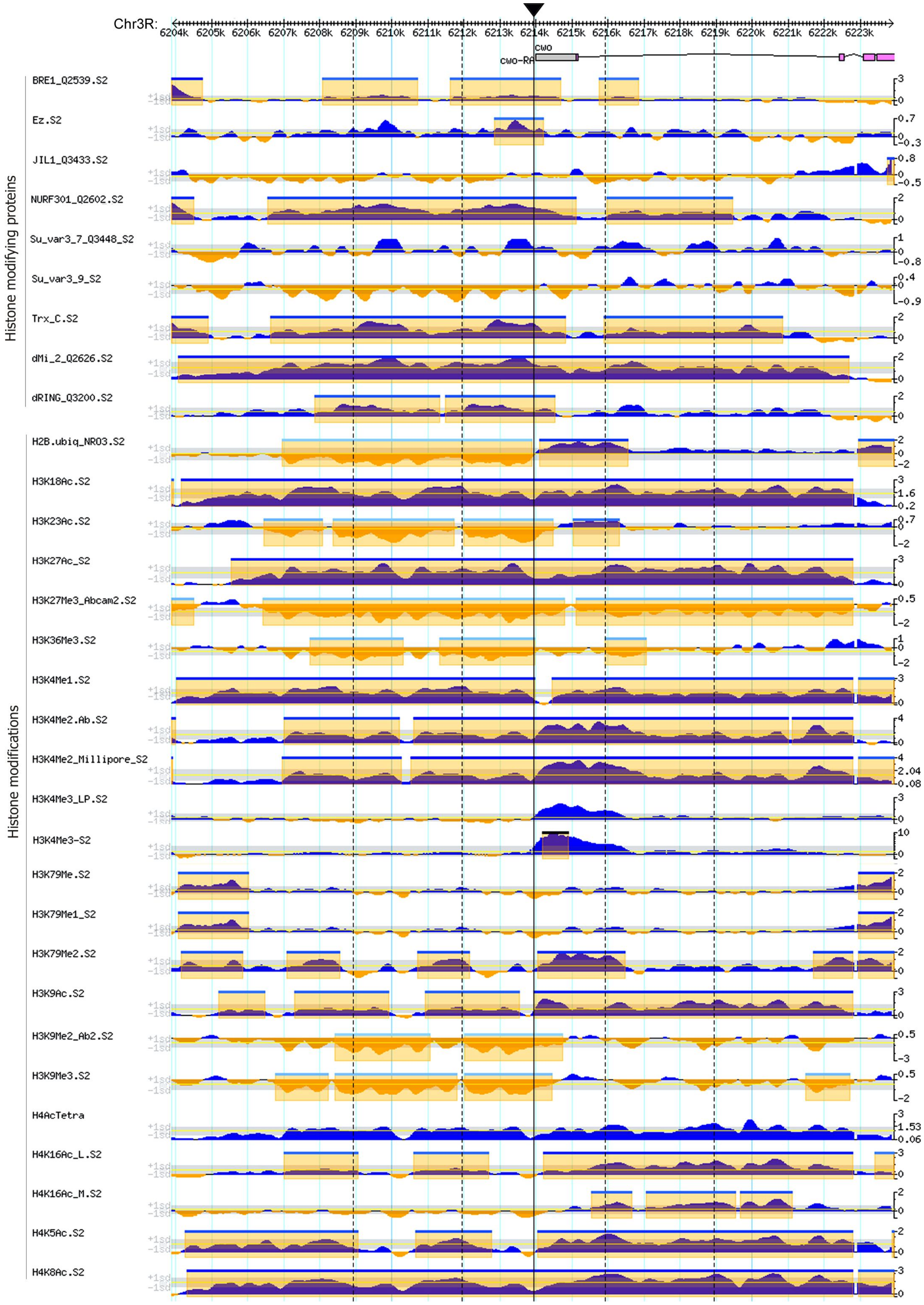
Chr3R:
6204k 6205k 6206k 6207k 6208k 6209k 6210k 6211k 6212k 6213k 6214k 6215k 6216k 6217k 6218k 6219k 6220k 6221k 6222k 6223k
cwo
cwo-RA
Histone modifying proteins
BRE1_Q2539.S2
Ez.S2
JIL1_Q3433.S2
NURF301_Q2602.S2
Su_var3_7_Q3448_S2
Su_var3_9_S2
Trx_C.S2
dMi_2_Q2626.S2
dRING_Q3200.S2
Histone modifications
H2B.ubiq_NRO3.S2
H3K18Ac.S2
H3K23Ac.S2
H3K27Ac_S2
H3K27Me3_Abcam2.S2
H3K36Me3.S2
H3K4Me1.S2
H3K4Me2.Ab.S2
H3K4Me2_Millipore_S2
H3K4Me3_LP.S2
H3K4Me3-S2
H3K79Me.S2
H3K79Me1_S2
H3K79Me2.S2
H3K9Ac.S2
H3K9Me2_Ab2.S2
H3K9Me3.S2
H4AcTetra
H4K16Ac_L.S2
H4K16Ac_M.S2
H4K5Ac.S2
H4K8Ac.S2
+1sd
-1sd

Region 87C8/C9

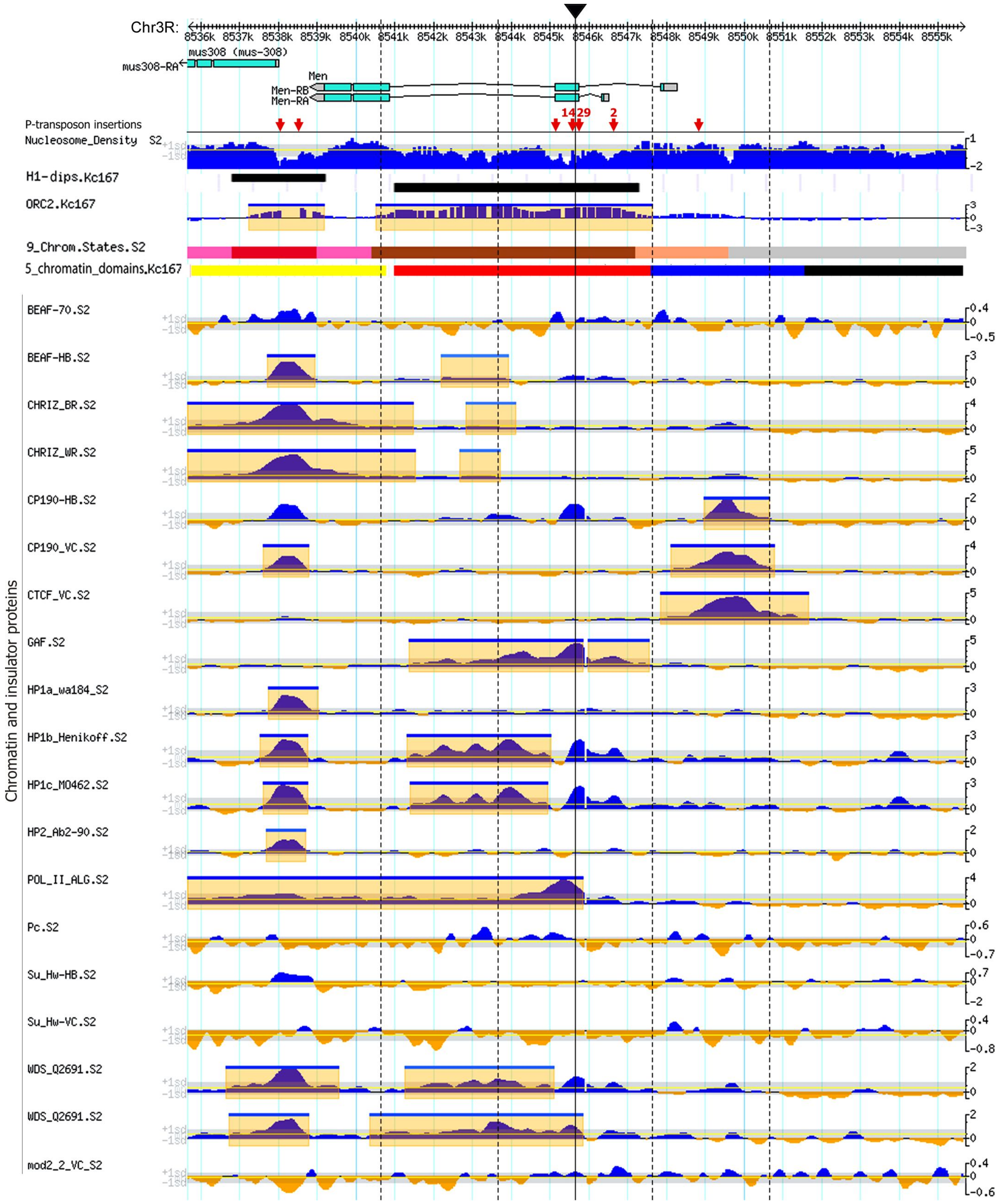

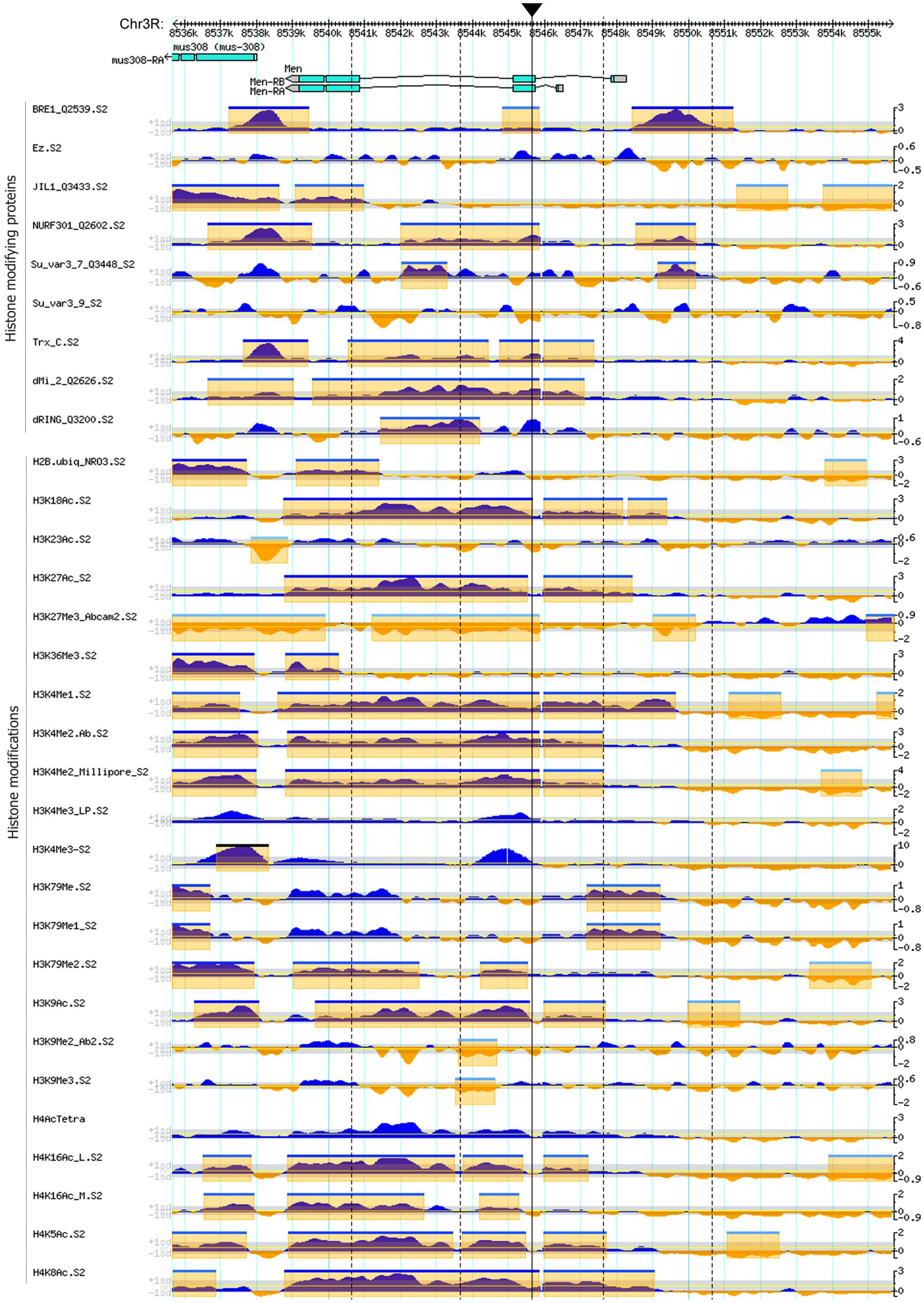
Chr3R:
8536k 8537k 8538k 8539k 8540k 8541k 8542k 8543k 8544k 8545k 8546k 8547k 8548k 8549k 8550k 8551k 8552k 8553k 8554k 8555k
mus308 (mus-308)
mus308-RA
Men
Men-RB
Men-RA
Histone modifying proteins
BRE1_Q2539.S2
Ez.S2
JIL1_Q3433.S2
NURF301_Q2602.S2
Su_var3_7_Q3448_S2
Su_var3_9_S2
Trx_C.S2
dMi_2_Q2626.S2
dRING_Q3200.S2
Histone modifications
H2B.ubiq_NRO3.S2
H3K18Ac.S2
H3K23Ac.S2
H3K27Ac_S2
H3K27Me3_Abcam2.S2
H3K36Me3.S2
H3K4Me1.S2
H3K4Me2.Ab.S2
H3K4Me2_Millipore_S2
H3K4Me3_LP.S2
H3K4Me3-S2
H3K79Me.S2
H3K79Me1_S2
H3K79Me2.S2
H3K9Ac.S2
H3K9Me2_Ab2.S2
H3K9Me3.S2
H4AcTetra
H4K16Ac_L.S2
H4K16Ac_M.S2
H4K5Ac.S2
H4K8Ac.S2
