# Supplementary figures and images for: Protein composition of interband regions in polytene and cell line chromosomes of Drosophila melanogaster
Source: BMC Genomics. 2011 Nov 18;12:566. doi: 10.1186/1471-2164-12-566 (PMC3240664; doi:10.1186/1471-2164-12-566)

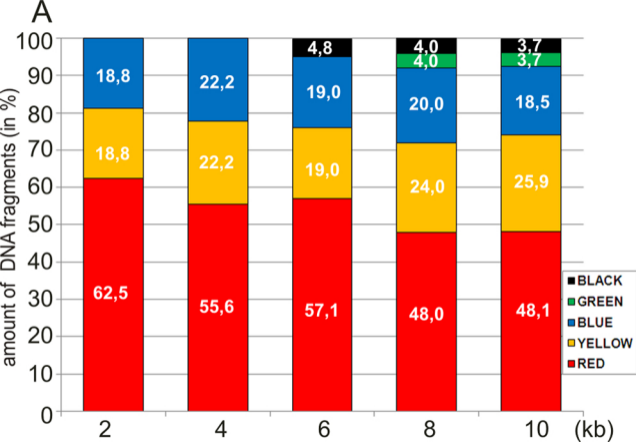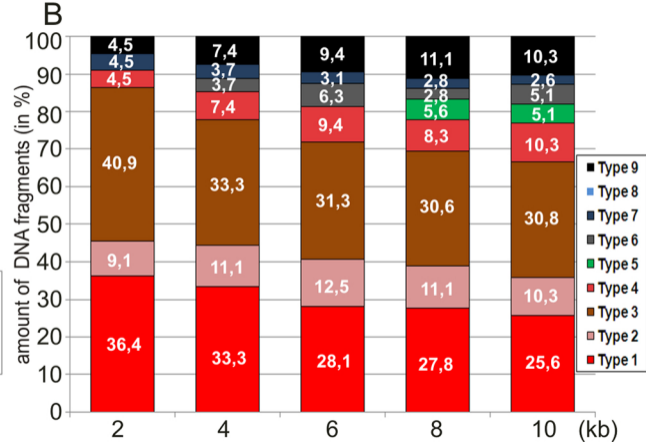

Supplement: Additional file 3 — Figure S2. Frequency of chromatin states in 13 regions of D. melanogaster genome that correspond to interbands in polytene chromosomes. A - 5 "colored" chromatin states according to [35]; B - 9 chromatin types according to [36]. Sizes of DNA segments centered at the insertion sites of reference P-transposons (X axis); Percentage of DNA fragments associated with a particular type of chromatin calculated for each segment (Y axis). [file 1471-2164-12-566-S3.PDF]
